# Molecular Camouflage of *Plasmodium falciparum* Merozoites by Binding of Host Vitronectin to P47 Fragment of SERA5

**DOI:** 10.1038/s41598-018-23194-9

**Published:** 2018-03-22

**Authors:** Takahiro Tougan, Jyotheeswara R. Edula, Eizo Takashima, Masayuki Morita, Miki Shinohara, Akira Shinohara, Takafumi Tsuboi, Toshihiro Horii

**Affiliations:** 1 Department of Molecular Protozoology, Research Institute for Microbial Diseases, Osaka University, 3-1 Yamadaoka, Suita, Osaka 565-0871 Japan; 2 Division of Malaria Research, Proteo-Science Center, Ehime University, 3 Bunkyo-cho, Matsuyama, Ehime 790-8577 Japan; 3 Department of Advanced Bioscience, Faculty of Agriculture, Kindai University, 3327-204 Nakamachi, Nara, Nara 631-8505 Japan; 4 Department of Integrated Protein Functions, Institute for Protein Research, Osaka University, 3-2 Yamadaoka, Suita, Osaka 565-0871 Japan

## Abstract

The malaria parasite *Plasmodium falciparum* proliferates in the blood stream where the host immune system is most active. To escape from host immunity, *P. falciparum* has developed a number of evasion mechanisms. Serine repeat antigen 5 (SERA5) is a blood stage antigen highly expressed at late trophozoite and schizont stages. The P47 N-terminal domain of SERA5, the basis of SE36 antigen of the blood stage vaccine candidate under clinical trials, covers the merozoite surface. Exploring the role of the P47 domain, screening of serum proteins showed that vitronectin (VTN) directly binds to 20 residues in the C-terminal region of SE36. VTN co-localized with P47 domain in the schizont and merozoite stages. Phagocytosis assay using THP-1 cells demonstrated that VTN bound to SE36 prevented engulfment of SE36-beads. In addition, several serum proteins localized on the merozoite surface, suggesting that host proteins camouflage merozoites against host immunity via binding to VTN.

## Introduction

Malaria is widespread in tropical and subtropical regions and despite substantial progress in malaria control, millions of people, particularly in Africa, remain at risk of disease and death^1^. Among five Plasmodium species that infect humans, *Plasmodium falciparum* is the most deadly species that cause half a million deaths annually. The parasite has developed a number of host immune evasion mechanisms that allow chronic and repeated infections. There are at least two-well studied mechanisms. One is genetic polymorphism of parasite surface antigens like circumsporozoite protein (CSP), apical membrane antigen-1 (AMA-1) and merozoite surface protein (MSP-1) (for review, ref.^2^). New infections bearing different polymorphic surface antigens escapes host immunity acquired from previous infection. Another is antigenic variation observed in erythrocyte membrane antigen 1 (EMA-1) and rifin (for review, ref.^3^). The expression of a member from a gene family occasionally changes presenting a different antigenic type on the red blood cell (RBC) surface. In addition to these, a wide range of mechanisms to evade host immune responses has been reported in other parasites (for review, ref.^4^). Some parasites avoid the host immune response by camouflage or adsorbing host proteins to its surface. For example, it has been suggested that *Trichomonas vaginalis* binds the human protein (CD59) for resistance to complement-mediated lysis^5^. A multifunctional schistosome Fc receptor protein (paramyosin or Pmy) was identified to bind to human Fc and C1q^6^, potentially interfering with the immune processes of Fc receptors and complement activation in schistosomes.

Serine repeat antigen 5 (SERA5)^7^ is an abundant blood stage antigen highly expressed at late trophozoite and schizont stages as a 120-kDa precursor and secreted into the lumen of the parasitophorous vacuole of infected red blood cells (iRBCs) after removal of the signal peptide. A recent paper implicated SERA5 as an important kinetic regulator of merozoite egress and suggested that a proteolytic cleavage during the schizont stage is essential for its function^8^. SERA5 is cleaved by a subtilisin-like serine protease called SUB1 to 47-, 56-, and 18-kDa fragments, corresponding to P47, P56, and P18 domains respectively^9^. P56 fragment is cleaved by an unknown protease to P50 and P6 fragments^10^. Among these fragments, P50 fragment has cysteine protease motif but its protease activity was denied^11^. Other domains, P47 and P18 fragments form a 65-kDa complex by disulfide bonding, and associates with the merozoite surface, suggesting that these fragments are exposed to human serum^9,12^. The function of the 65-kDa complex remains poorly understood. However, recombinant proteins made from P47 domain has been shown to be immunogenic in the field and induced antibodies inhibited *in vitro* and in animal models erythrocyte invasion and parasite replication^13^ (for review, ref.^14^).

We have been developing SE36 antigen based on a modified P47 domain of SERA5. SE36 is the recombinant P47 without the polyserine repeats^13,15^. SE36 antigen was formulated with aluminum hydroxyl gel (AHG) as a malaria vaccine candidate (BK-SE36) under Good Manufacturing Practice (GMP). Phase Ia clinical trial of SE36/AHG in healthy Japanese adult volunteers showed 100% sero-conversion rate and no serious adverse events^13^. In Phase Ib clinical trial for ages 6–20 years-old (Stage 2), immunogenicity of SE36 antigen was high only in younger cohorts in Uganda. Nevertheless, the one year follow-up clinical research of Stage 2, showed that BK-SE36 vaccination reduced clinical malaria by 72% compared with the control group^16^. *In vitro* studies demonstrated that antibodies against the P47 domain showed antiparasitic effects such as complement-mediated cell lysis of segmented schizont^17^ or merozoite agglutination^18^. Antibody-dependent cellular inhibition (ADCI) assay revealed that anti-SE36 IgG from Ugandan adult serum inhibited parasite growth^15,19^.

Vitronectin (VTN) is a multifunctional protein containing several binding motifs and associates to various proteins and glycans^20,21^. Highly glycosylated (about 30% carbohydrate by weight), it is an abundant (0.3 mg/mL) and multi-functional protein that interacts with a wide variety of proteins inducing conformational changes and acts as key regulator for tissue regeneration or remodelling^22^. Originally described as an inhibitor of complement-mediated cytolysis, VTN modulates the lytic activity of complement by interacting with constituents of the membrane attack complex (MAC)^23^. And VTN interacts with VTN receptor (α _v_β_3_ integrin) through Arg-Gly-Asp (RGD) motif. The RGD motif enhances monocyte-induced phagocytosis of apoptotic target cells opsonized with IgG or complement C3b^24,25^. On the other hand, it was reported that VTN inhibits efferocytosis by binding to both phagocytes and apoptotic target cells^26^.

In this study, we show that VTN directly binds to the C-terminal region of SE36 and that VTN is internalized into iRBCs and co-localizes with P47 fragment on the merozoite surface. We also observed that VTN binding in merozoite-mimic beads prevented the beads engulfment by THP-1 cells. In addition, serum proteins that bind to VTN also covers the merozoite surface.

## Results

### Identification of SE36-binding protein(s)

To see whether P47 fragment could bind to the bulk of serum proteins, we conducted ELISA-based binding assay using SE36, a recombinant form of P47 domain. The assay between SE36 and total serum protein in naïve human serum (NHS) with double reciprocal dilutions suggested that SE36 binds to serum protein(s) (Supplementary Fig. 1). To obtain SE36-binding proteins in NHS, NHS was applied to the SE36-immobilized column and then SE36-binding proteins were eluted. The eluates were subjected to shotgun Liquid chromatography–tandem mass spectrometry (LC-MS/MS) analysis. The comparison of two fractions (eluates from SE36 and control columns, Supplementary Fig. 2) revealed that several complement factors (n = 8/66) and apolipoproteins (n = 8/66) are especially enriched (Supplementary Table 1).

### VTN directly binds to SE36

**Figure 1:**
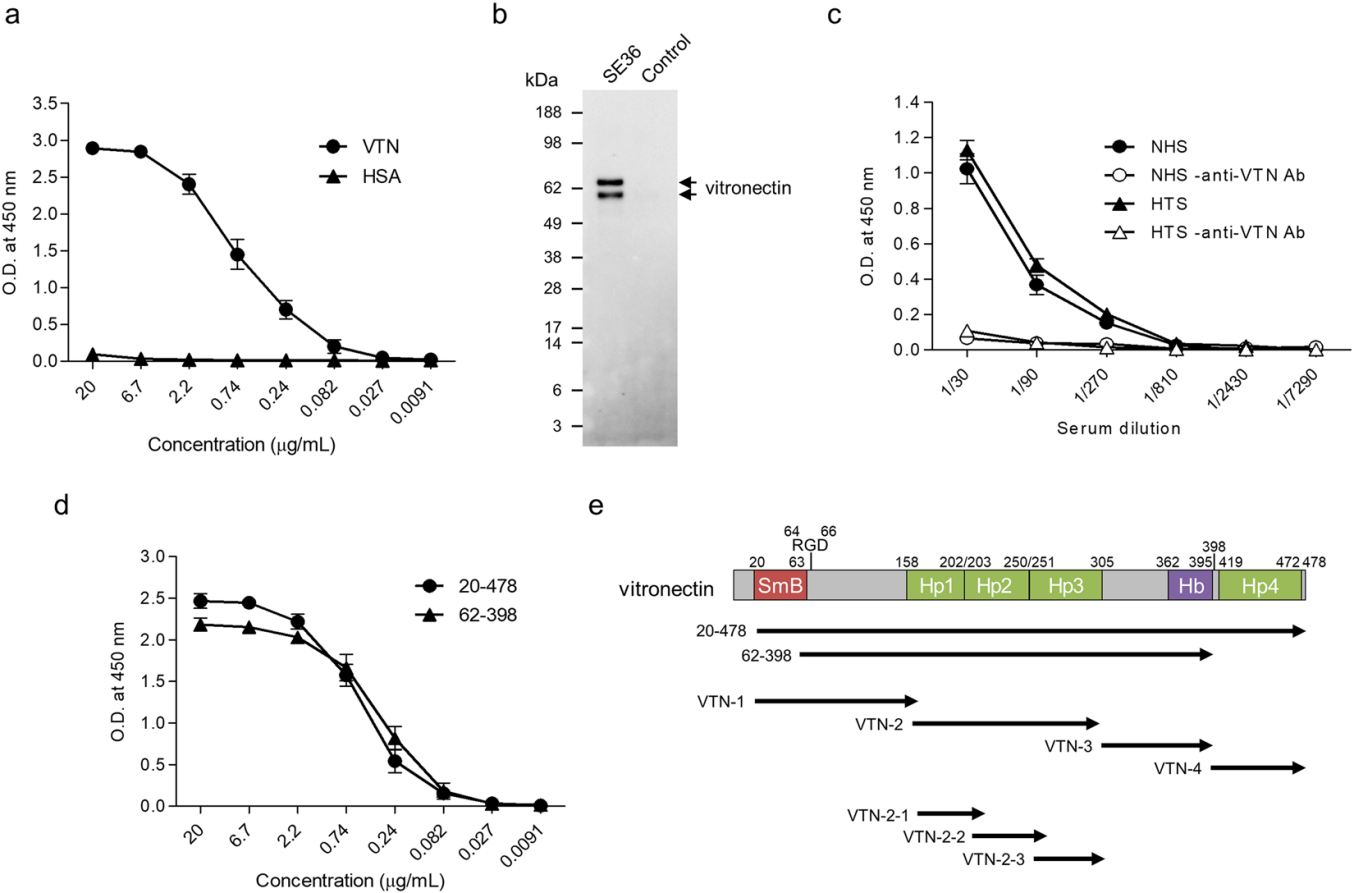
Binding assay of vitronectin (VTN) to SE36. (**a**) Reactivity of the purified VTN and HSA against SE36. SE36 was adsorbed to microtiter plate and VTN or human serum albumin (HSA) was added at the indicated concentrations. (**b**) Western blotting of elutes from SE36-immobilized and control columns. The anti-VTN pAb detected two forms (V75 and V65; upper band and lower band, respectively) of VTN. Each elute was run in an SDS-PAGE gel and probed with anti-VTN pAb (diluted 1:2000). (**c**) Reactivity of VTN in naïve human serum (NHS) and Ugandan high anti-SE36 IgG-titer serum (HTS) against SE36. SE36 was adsorbed to microtiter plate and NHS or HTS was added at the indicated dilutions (closed symbols). Open symbols show reactions without anti-VTN Ab as negative control. (**d**) Reactivity of commercially available VTN recombinants (see also Fig. 1e) against SE36. SE36 was adsorbed to microtiter plate and VTN recombinants were added at the indicated concentrations. (**e**) Schematic representation of VTN. “SmB”, “Hp”, and “Hb” indicate Somatomedin-B motif, Hemopexin domain, and Heparin-binding region, respectively. The number 398 denotes the endogenous cleavage site. The designations “20–478” and “62–398” indicate commercially available VTN recombinants. VTN-1 to -4, as well as VTN-2-1 to -2-3 denote truncated recombinant forms. Results in (**a**, **c**, and **d**) are expressed as means ± SD from three independent experiments. In (**c**), statistical analysis between NHS and HTS was performed using a Mann-Whitney U test. No significant differences were found.

To identify protein(s) that directly binds to SE36, we performed an ELISA-based direct binding assay using SE36 and each purified human protein. No obvious direct binding was observed for complement factors (C5, C7, C8, C9, and H), apolipoproteins (ApoAI, HDL, and LDL), thrombin, clusterin, fibronectin, serum albumin, CD5L, or CD14 (Supplementary Fig. 3). Nonetheless, purified VTN but not human serum albumin (HSA) bound to SE36 in a concentration-dependent manner (Fig. 1a). And VTN in NHS was adsorbed to SE36-immobilized column and eluted (Fig. 1b). VTN in NHS and Ugandan high anti-SE36 IgG-titer serum (HTS) equally bound to SE36 despite the existence of other serum proteins (Fig. 1c). This similar binding capacity of VTN in NHS and HTS suggest that naturally acquired anti-SE36 IgG in HTS does not inhibit the binding of VTN. Moreover, two commercially available recombinant forms of VTN equally bound to SE36, suggesting that both Somatomedin-B motif (SmB) and Hemopexin domain 4 (Hp4) on VTN may not be essential for the binding. Since the recombinant VTN (62–398) produced in *E. coli* was not glycosylated, yet directly bound to SE36, the glycosylation moiety on VTN may, likewise, not be necessary for the binding (Fig. 1d,e). From above results, VTN directly binds to SE36 even in the presence of other serum proteins as well as under presence of naturally acquired anti-SE36 IgG.

### Binding site mapping of SE36 to VTN

**Figure 2:**
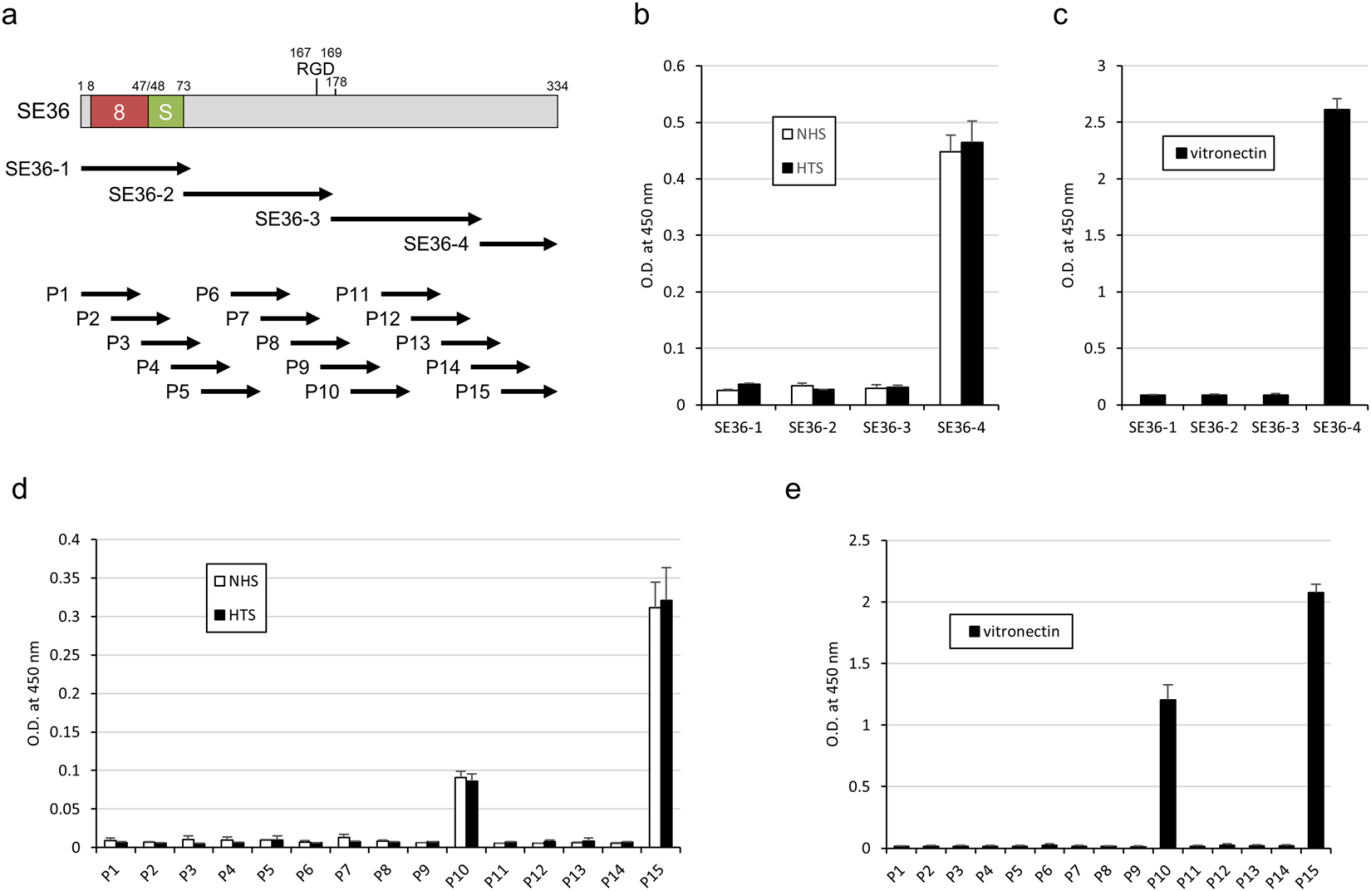
Mapping of binding site of VTN in SE36. (**a**) Schematic representation of SE36. The “8” and “S” indicate octamer repeat and serine rich region, respectively. “RGD” indicates RGD motif. The number 178 denotes the position of polyserine sequence present in PfSERA5 but deleted in SE36^13^. SE36-1 to -4 denote truncated recombinant forms. P1 to P15 are synthetic peptides^19^. (**b**) Reactivity of VTN in NHS and HTS against each truncated SE36 recombinant. Each recombinant was adsorbed to microtiter plate and NHS and HTS were added at 1:2000 dilution. (**c**) Reactivity of the purified VTN against each truncated SE36 recombinant. Each recombinant was adsorbed to microtiter plate and the purified VTN was added at 2 μg/mL concentration. (**d**) Reactivity of VTN in NHS and HTS against each synthetic peptide. Each synthetic peptide was adsorbed to microtiter plate (0.3 μM) and NHS and HTS were added at 1:2000 dilution. (**e**) Reactivity of the purified VTN against each synthetic peptide. Each synthetic peptide was adsorbed to microtiter plate and the purified VTN was added at 2 μg/mL concentration. Results in (**b**–**e**) are expressed as means ± standard deviation (SD) from three independent experiments. In (**b** and **d**), statistical analyses between NHS and HTS were performed using a Mann-Whitney U test. No significant differences were found.

To define the VTN-binding site in SE36, truncated SE36 recombinants, SE36-1 to -4, were constructed and purified (Fig. 2a and Supplementary Fig. 4). The binding assay clearly demonstrated that VTN in NHS and HTS exclusively bind to SE36-4 (Fig. 2b). This binding was further confirmed using purified VTN (Fig. 2c). Mapping the binding site with 15 overlapping peptides of SE36 showed that serum VTN remarkably binds to peptide-15 and modestly to peptide-10 (Fig. 2a,d). This binding was confirmed with the purified VTN (Fig. 2e). The binding to peptide-10 was thought to be non-specific, since VTN did not bind to SE36-3 that includes the peptide-10 sequence (Fig. 2a–c). Moreover, from our experience, peptide-10 bound to many proteins non-specifically (data not shown). VTN binding to peptide-15 corresponds to 40 residues (NH_2_-Phe-Asn-Ile-Glu-Lys-Cys-Phe-Gln-Cys-Ala-Leu-Leu-Val-Glu-Lys-Glu-Asn-Lys-Asn-Asp-Val-Cys-Tyr-Lys-Tyr-Leu-Ser-Glu-Asp-Ile-Val-Ser-Asn-Phe-Lys-Glu-Ile-Lys-Ala-Glu-COOH) of the C-terminal region on SE36. The similar binding profile of VTN in NHS and HTS to SE36-4 and peptide-15 (Fig. 2b,d) suggest, once more, that naturally acquired anti-SE36 IgG does not disturb the binding as shown in Fig. 1c.

### Localization of VTN

**Figure 3:**
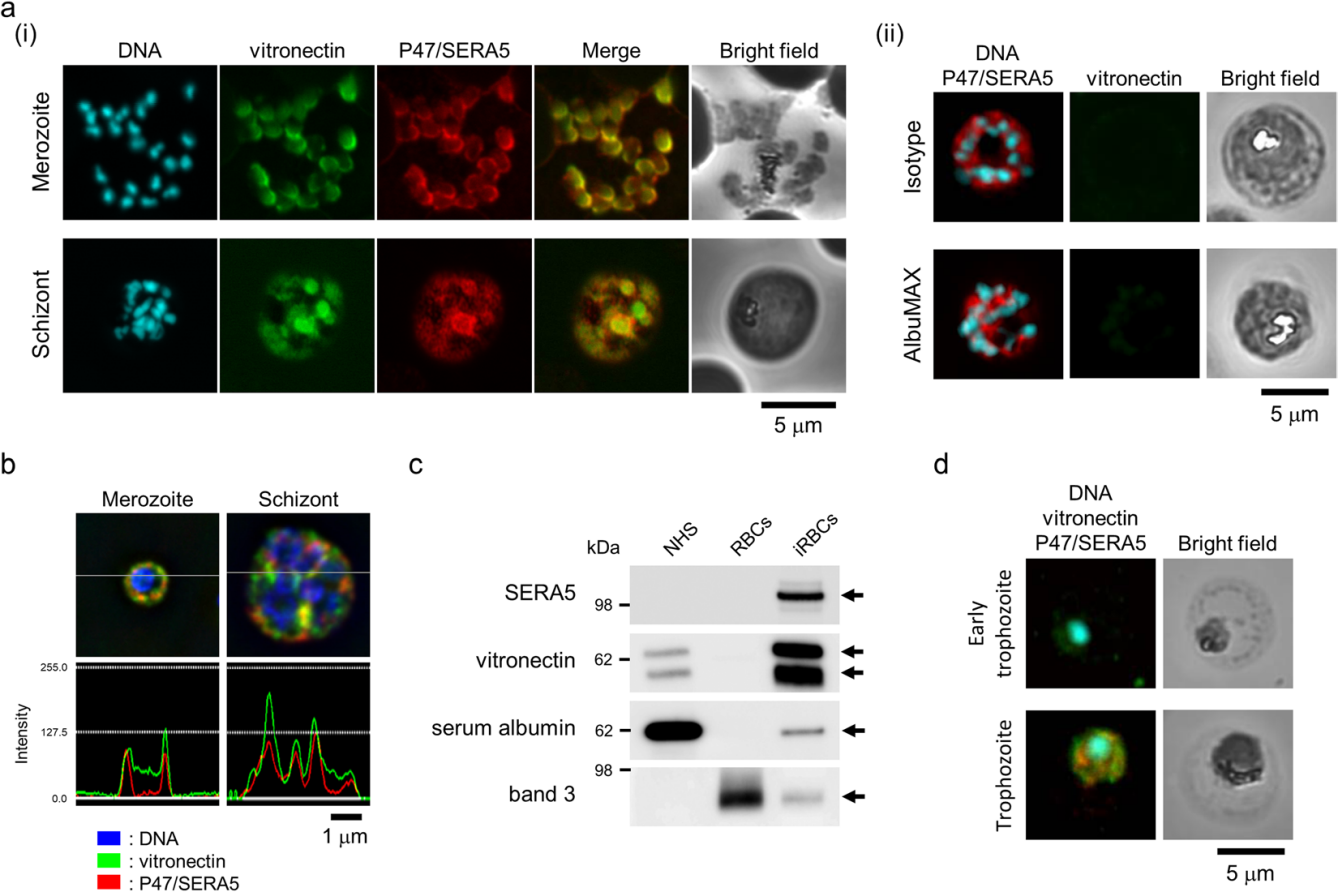
Localization of VTN and P47 domain/SERA5. (**a**) (i) Representative immunofluorescence assay (IFA) images of merozoites and schizont. (ii) *Upper panels*, Representative IFA images of schizont. VTN was probed with an isotype control mAb instead of anti-VTN mAb. *Lower panels*, Representative IFA images of schizont cultured in AlbuMAX-I medium. Target proteins were probed with anti-VTN mAb (green), anti-SE36 rabbit serum (red), and DAPI nuclear stain (blue). Scale bar, 5 μm. (**b**) Representative IFA images of merozoite and schizont stages under deconvolution microscopy. Probes used were the same as in panel a. Upper panels show the localization of P47 domain/SERA5 during merozoite and schizont stages. Lower panels show the intensity of green and red signals at the section of the gray line in the upper panel. Scale bar, 1 μm. (**c**) Western blotting of NHS, red blood cell (RBC) lysate, and iRBC lysate. The blots were probed with anti-SE36 mouse serum (diluted 1:1000), anti-VTN pAb (diluted 1:2000), anti-albumin pAb (diluted 1:2000), and anti-band 3 mAb (diluted 1:2000). Band 3 is RBC-specific protein and used as loading control. (**d**) Representative IFA images of early trophozoite and trophozoite. Target proteins were probed with anti-VTN mAb (green), anti-SE36 rabbit serum (red), and DAPI nuclear stain (blue). Scale bar, 5 μm.

Confocal microscopy showed that VTN co-localizes with P47 fragment on the merozoite surface (Fig. 3a(i), upper panels). We also observed that VTN co-localized with P47 domain/SERA5 in iRBCs during the schizont stage (Fig. 3a(i), lower panels), and was absent in healthy non-infected RBCs (see also Fig. 3c and ref.^27^). No green signal of VTN was observed with isotype control (Fig. 3a(ii), upper images) and with iRBCs cultured in AlbuMAX-I medium instead of human serum (Fig. 3a(ii), lower images). Images from deconvolution microscopy displayed the co-localization of both proteins during the merozoite and schizont stages (Fig. 3b, upper panels). This co-localization was confirmed by overlapping intensities of green and red fluorescence at the section of gray lines (Fig. 3b: lower panels). Western blotting showed that VTN was detected in the iRBC lysate but not in the RBC lysate (Fig. 3c). The internalization of VTN was detected in the early trophozoite stage, when SERA5 was not yet expressed (Fig. 3d). At trophozoite stage, it colocalizes with SERA5. From above, VTN co-localizes with P47 domain/SERA5 in iRBCs and with P47 fragment on the merozoite surface.

### Binding of bivalent VTN to SE36

**Figure 4:**
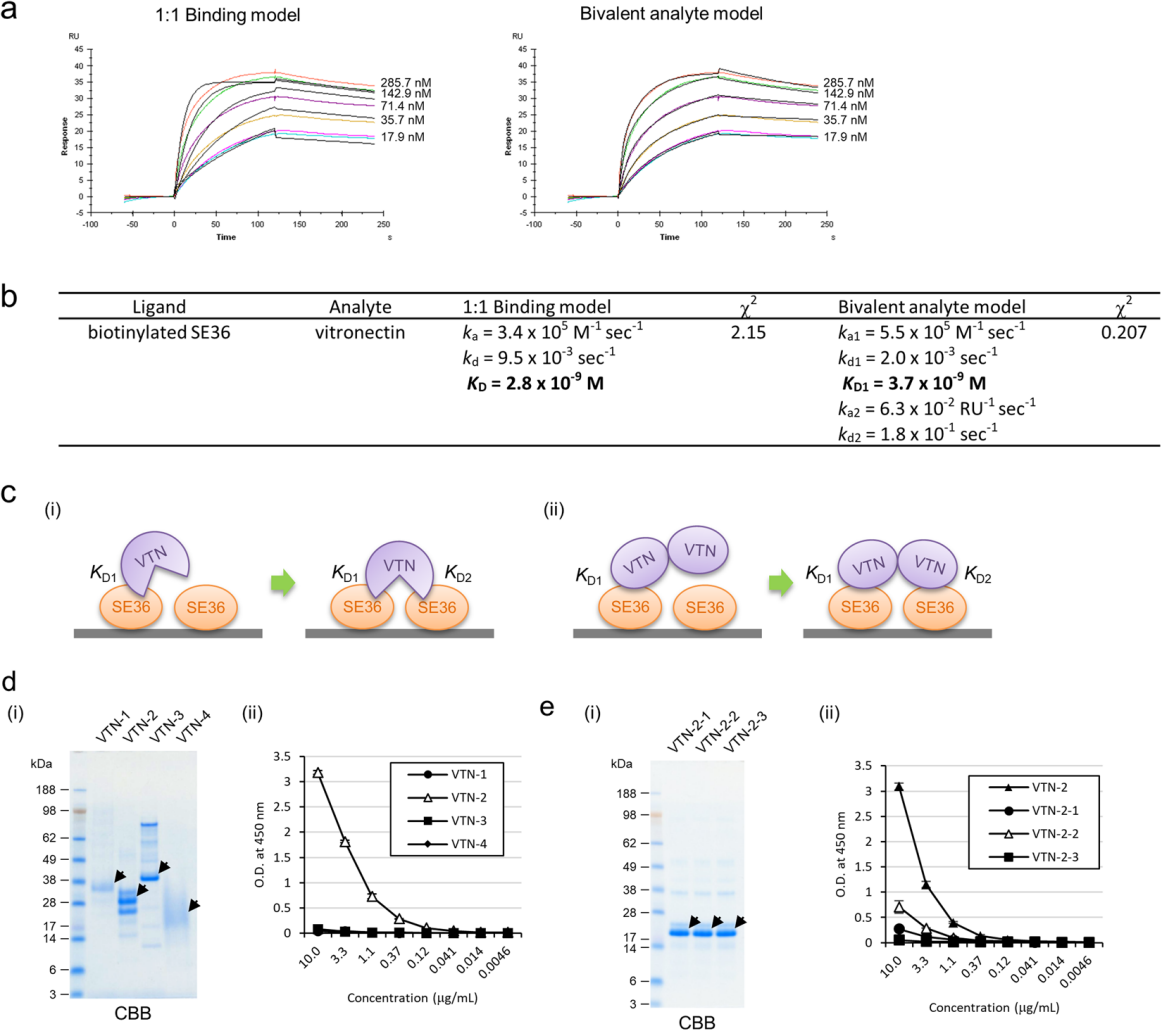
Characterization of VTN binding to SE36. (**a**) Biophysical interaction analysis of the binding of VTN to SE36 using surface plasmon resonance (SPR). The raw data (color lines) were fitted to 1:1 binding and bivalent analyte model (black lines) using the Biacore T200 Evaluation software. The fit parameters from these models are summarized in (**b**). These experiments were performed in triplicate, and representative data are shown. (**b**) Kinetic and equilibrium constants for the binding of VTN to SE36. (**c**) Schematic representation of two schemes in bivalent binding model. (i) VTN contains two binding sites. (ii) VTN contains a single binding site and forms a dimer. (**d**) (i) Purity of VTN-1 to -4 recombinants confirmed by sodium dodecyl sulphate-polyacrylamide gel electrophoresis (SDS-PAGE) and Coomassie Brilliant Blue (CBB) staining. Two micrograms of each recombinant was run in a gel. Arrows indicate target proteins. Since estimated molecular weights were 18, 21, 14, and 12 kDa in VTN-1 to -4, respectively, it was presumed that these recombinants were glycosylated in *Pichia pastoris*. (ii) Reactivity of SE36 against each truncated VTN recombinant. Each recombinant was adsorbed to microtiter plate (1 μg/mL) and SE36 was added at the indicated concentrations. (**e**) (i) Purity of VTN-2-1 to -2-3 recombinants confirmed by SDS-PAGE and CBB staining. Two micrograms of each of recombinant was run in a gel. Arrows indicate target proteins. Since estimated molecular weights were 8 kDa in VTN-2-1 to -2-3, it was, likewise, presumed that these recombinants were glycosylated in *P. pastoris*. All recombinant proteins of VTN (**d** and **e**) were detected by anti-His tag antibody in western blotting and was purified by His GraviTrap. (ii) Reactivity of SE36 against each truncated VTN recombinant. Each recombinant was adsorbed to microtiter plate (1 μg/mL) and SE36 was added at the indicated concentrations.

To characterize physicochemical properties of the interaction between SE36 and VTN, kinetics analysis was implemented by surface plasmon resonance (SPR). Kinetic measurement showed concentration-dependent binding of VTN to SE36 (Fig. 4a). The fitting analysis of acquired sensorgrams revealed that the fitting to the bivalent analyte model (γ^2^ = 0.207) is better than that of the simple 1:1 binding model (γ^2^ = 2.15) (Fig. 4a,b). In the bivalent analyte model, the first interaction has an association rate constant (*k*_a1_) of 5.5 × 10^5^ M^−1^ sec^−1^ and a dissociation rate constant (*k*_d1_) of 2.0 × 10^−3^ sec^−1^, yielding an equilibrium dissociation constant (*K*_D1_) of 3.7 × 10^−9^ M (Fig. 4b).

### Binding site mapping of VTN to SE36

The bivalent analyte model implicates two different binding forms of VTN; (i) single VTN has two binding sites and (ii) VTN having single binding site forms homodimer (Fig. 4c). To define the binding form of VTN, a binding site in VTN was explored using truncated VTN recombinants, VTN-1 to -4 (Fig. 1e). The binding assay demonstrated that VTN-2 remarkably binds to SE36 (Fig. 4d). VTN-2 contains three hemopexin motifs and further binding assay was performed using VTN-2-1 to -2-3 (Fig. 1e). The assay indicated that the central region of VTN corresponding to VTN-2-2 especially binds to SE36 (Fig. 4e). In addition, previous report implicated that VTN forms dimer and even multimer and is enriched in extracellular matrix of endothelial cells^28^. These findings suggest that VTN with single binding site forms a homodimer and binds to SE36 as shown in Fig. 4c(ii).

### Phagocytosis assay

**Figure 5:**
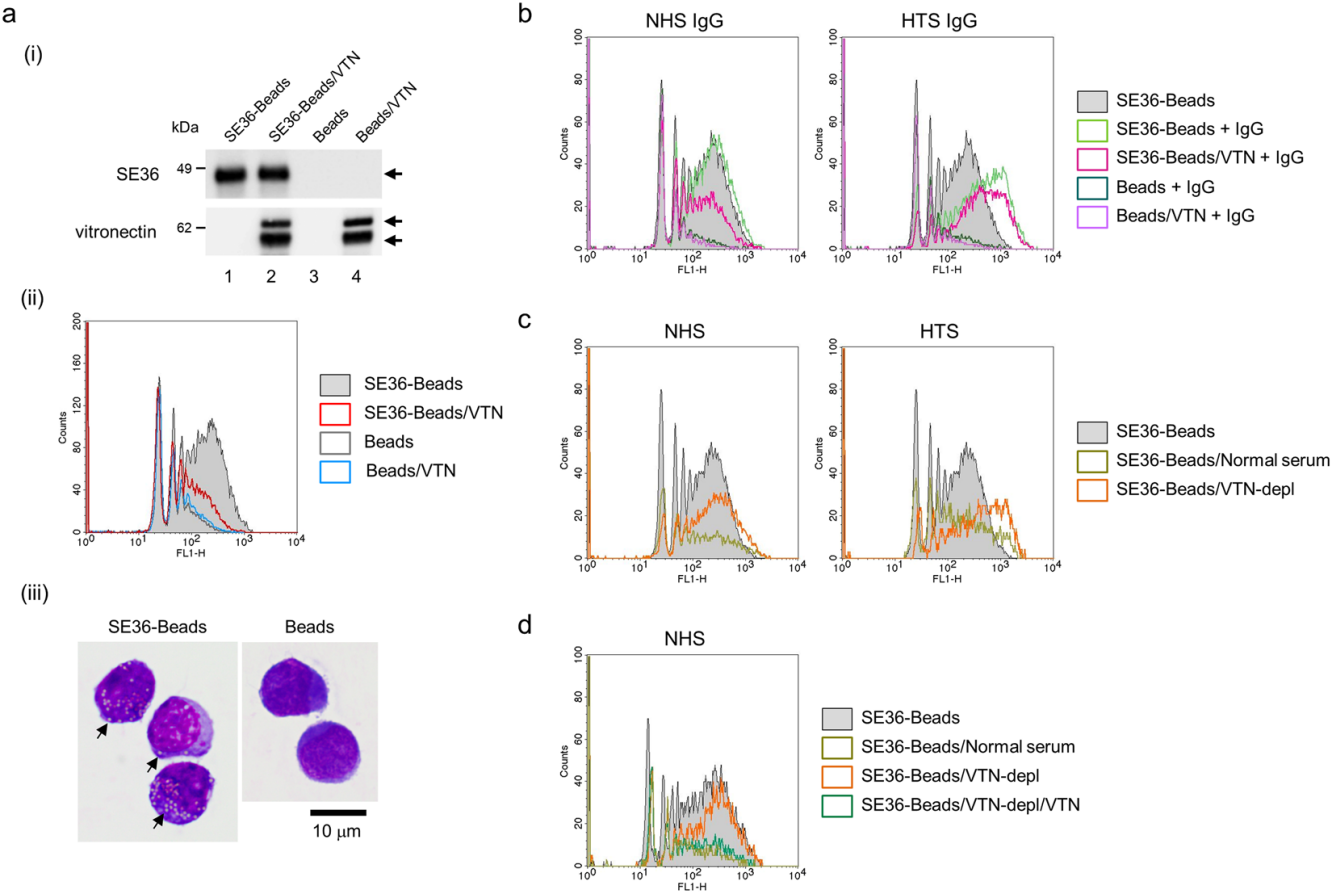
Phagocytosis assay by THP-1 cells. (**a**) (i) Western blotting of bound protein(s) on latex beads after solubilization. Lane 1: SE36-beads, lane 2: SE36-beads with VTN, lane 3: latex-beads alone, lane 4: beads with VTN. SE36 and VTN bound to the beads were detected by anti-SE36 mouse serum (diluted 1:1000) and anti-VTN pAb (diluted 1:2000), respectively. Arrows indicate the target proteins. (ii) Representative flow cytometry (FCM) histogram of the antibody-independent engulfment of SE36-beads with or without VTN by THP-1 cells. (iii) Giemsa staining of engulfed SE36-beads (left panel) and non-treated beads (right panel) by THP-1 cells. Arrows indicate the engulfed SE36-beads. Scale bar, 10 μm. (**b**) FCM histogram of the engulfment by THP-1 cells of SE36-beads with or without VTN and/or purified IgG from NHS or HTS. (**c**) FCM histogram of the engulfment by THP-1 cells of SE36-beads treated with normal serum or VTN-depleted serum (VTN-depl). (**d**) FCM histogram of the engulfment by THP-1 cells of SE36-beads with or without VTN-depl, with or without purified VTN or with normal serum.

Previous reports demonstrated phagocytosis of merozoite by human peripheral blood mononuclear cells and THP-1 monocytes^29,30^. To elucidate the role of VTN on the merozoite surface, SE36-beads with or without VTN acted as a merozoite models. The coating of SE36 and the binding of VTN were confirmed by western blotting and confocal microscopy (Fig. 5a(i) and Supplementary Fig. 5). Unexpectedly, VTN non-specifically bound to latex beads (Fig. 5a(i), lower panel, lane 4). The phagocytosis assay revealed that SE36-beads are engulfed by THP-1 cells in an antibody-independent manner (Fig. 5a(ii), phagocytosis index (PI): 100%; (iii) and Supplementary Movie 1). This engulfment was observed in SE36-beads but not in other proteins such as thrombin-coated beads (Supplementary Fig. 6, PI: 46.1%) and was seen with actin polymerization (Supplementary Fig. 7). The engulfment was inhibited by VTN bound to SE36-beads (Fig. 5a(ii), red line; PI: 79.9%). SE36 bound to THP-1 cells and competed with VTN (Supplementary Fig. 8), suggesting that THP-1 cells have uncharacterized receptor against SE36. Since IgG-dependent phagocytosis is commonly known (*e.g.* ref.^29^), we examined the influence of IgG purified from NHS or HTS on this engulfment. PIs are in Table 1. IgG purified from NHS did not affect both the engulfment and VTN-dependent inhibition of the engulfment (Fig. 5b, left panel). On the other hand, VTN suppressed the engulfment of SE36-beads even when the IgG from HTS enhanced the engulfment (Fig. 5b, right panel). These results suggested that VTN inhibits the engulfment of SE36-beads in an IgG-independent manner. To confirm the inhibition efficacy of serum VTN, we prepared VTN-depleted sera (Supplementary Fig. 9). After incubating SE36-beads with these sera, the binding of VTN on SE36-beads was confirmed by western blotting (Supplementary Fig. 10a). The engulfment of SE36-beads treated with normal serum was inhibited (Fig. 5c, dark lime lines), whereas this inhibition was partially recovered with VTN-depleted sera (Fig. 5c, orange lines). Further, when SE36 beads were treated with VTN-depleted NHS with purified VTN, the engulfment was inhibited (Fig. 5d, teal line) similar to SE36-beads treated with normal serum (Fig. 5d, dark lime line). The binding or unbinding of VTN to SE36-beads treated with VTN-depleted/VTN-supplemented sera was also confirmed by western blotting (Supplementary Fig. 10b). From these results we deduce that VTN inhibits the engulfment of SE36-beads, suggesting that merozoites with VTN evades phagocytosis.

**Table 1:** Observed phagocytosis indexes in THP-1 cells that engulfed SE36-Beads under the various treatments.

		Tested beads	Phagocytosis Index (%)
Fig. 5a(ii)		SE36-Beads	100.0
		SE36-Beads/VTN	79.9
		Beads	31.1
		Beads/VTN	32.8
Fig. 5b	NHS IgG	SE36-Beads	100.0
		SE36-Beads + IgG	131.0
		SE36-Beads/VTN + IgG	70.4
		Beads + IgG	24.4
		Beads/VTN + IgG	19.8
	HTS IgG	SE36-Beads	100.0
		SE36-Beads + IgG	291.6
		SE36-Beads/VTN + IgG	286.4
		Beads + IgG	31.1
		Beads/VTN + IgG	25.1
Fig. 5c	NHS	SE36-Beads	100.0
		SE36-Beads/Normal serum	70.1
		SE36-Beads/VTN-depl	162.5
	HTS	SE36-Beads	100.0
		SE36-Beads/Normal serum	86.6
		SE36-Beads/VTN-depl	266.6
Fig. 5d	NHS	SE36-Beads	100.0
		SE36-Beads/VTN-depl	140.7
		SE36-Beads/VTN-depl/VTN	49.6
		SE36-Beads/Normal serum	38.4

VTN, vitronectin; NHS, naïve human serum; HTS, Ugandan high anti-SE36 IgG-titer serum; and VTN-depl, VTN-depleted serum.

### Localization of serum proteins bound to VTN on the merozoite surface

**Figure 6:**
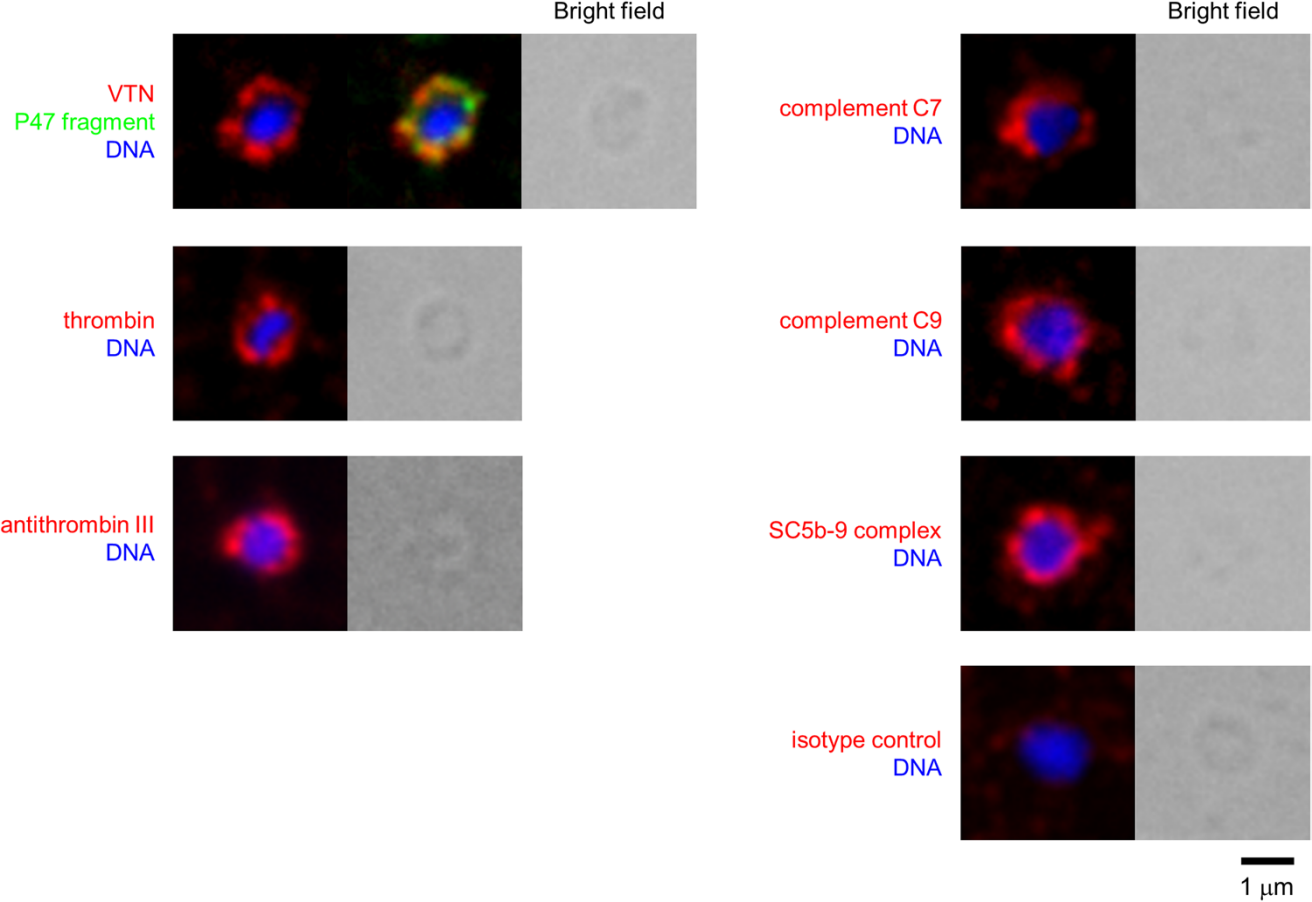
Localization of serum proteins that bind to VTN. IFA images showing the localization of VTN, thrombin, antithrombin III, complement C7, complement C9, SC5b-9 complex (red), P47 fragment (green), and DNA (blue) on the merozoite. Scale bar, 1 μm.

It was reported that VTN binds to thrombin–antithrombin III complex^31^ and terminal complement complex, C5b-7 complex and C9^22,32^. Fluorescent microscopy showed that thrombin, antithrombin III, complement C7, complement C9, and SC5b-9 complex, as well as VTN, localized on the merozoite surface (Fig. 6). However, these serum proteins, except VTN, did not show direct binding to SE36 as mentioned above (Supplementary Fig. 3). In VTN-depleted serum, binding of thrombin, complement C9, and full length of C7 was not observed or of significantly lower intensity (Supplementary Fig. 9b), suggesting that the recruitment of these proteins on the merozoite surface are facilitated by their interaction with VTN. Of interest is that the N-terminal domain of SERA5 (P47/SE36) binds to VTN and this interaction aids in the binding of host factors, likely aiding in merozoite escape and evasion of clearance by phagocytosis.

## Discussion

We found SE36-associated proteins in NHS (Japanese health adults with no exposure to malaria) by SE36-immobilized column chromatography followed by shotgun LC-MS/MS. The SE36-immobilized column retained a number of proteins that directly and indirectly associate with SE36. Among these proteins, VTN directly bound to SE36. VTN is a multifunctional protein containing several binding motifs that associate to various proteins and glycans. Based on the results we obtained, it is speculated that any SE36-binding proteins identified here bound to SE36 via VTN. Previous reports demonstrated that VTN has a binding domain for thrombin–antithrombin III complex^31^. It was also reported that VTN binds to C5b-7 complex and C9, interfering with the cytolytic action of MAC^22,32^. The hemopexin domain was also suggested to facilitate binding to a variety of proteins^21^.

Mapping the binding site in SE36 indicated that VTN binds to peptide-15 but not peptide-14 (Fig. 2d,e). Thus, VTN binds specifically to 18 residues (NH_2_-Tyr-Lys-Tyr-Leu-Ser-Glu-Asp-Ile-Val-Ser-Asn-Phe-Lys-Glu-Ile-Lys-Ala-Glu-COOH) at the far end of the C-terminal region of SE36 which do not overlap with peptide-14 (Fig. 2a). Sequence analysis of 445 geographically distributed *P. falciparum* parasites from nine countries in Africa, Southeast Asia, Oceania, and South America showed one genetic polymorphism, “Asn” to “Lys” at 11^th^ residue, in the 18 residues^33^, suggesting that the binding property of VTN is almost conserved in worldwide parasites. Moreover, we previously reported that the far end of the C-terminal region of peptide-15 forms α-helix structure^15^ but we could not find any structural similarity in the above mentioned VTN-associated proteins (data not shown). This may suggest that the binding of VTN to P47 fragment is not disturbed by other host factors.

In binding assays, VTN in NHS and HTS similarly bound to SE36, SE36-4, and peptide-15 (Figs 1c and 2b,d). The amount of VTN in NHS and HTS was almost equal (Supplementary Fig. 10), suggesting that behavior of VTN in both sera is almost the same and binding was not disturbed by naturally acquired anti-SE36 IgG. The RGD motif did not contribute to this binding, because VTN did not bind to peptide-8 and SE36-2 containing the RGD motif (Fig. 2). Furthermore, Stockmann *et al*. reported that vitronectin forms multimer in a concentration-dependent manner^28^. We used NHS and HTS diluted 1:100 (~3 μg/mL VTN), and purified and recombinant VTN (2 μg/mL) in the ELISA-based binding assay (Fig. 2), and purified VTN (20, 10, 5, 2.5, and 1.25 μg/mL) in the SPR assay (Fig. 4a). These concentrations were lower than the concentration used for multimer formation shown in the previous study^28^, thus we infer that protomer, especially dimer, but not multimer bound to SE36 as shown in the SPR assay (Fig. 4).

We demonstrated that VTN exists in iRBCs during schizont stage and on the merozoite surface (Fig. 3) likely functioning for evasion from host’s immune response (Figs 5 and 6). In *P. falciparum*, only few studies have reported on serum proteins that are incorporated into iRBCs^34–36^. Among these proteins, two proteins, kininogen^35^ and plasminogen^36^ were indicated to modulate host physiology. Most studies on the incorporation and the utilization of host factors have focused on low molecular weight compounds such as lipids and vitamins, and ions (for review, ref.^37^). Further analysis on the incorporation of VTN will provide new insights especially in consideration of the current study limitations, *e.g.*, use of latex beads for parasite phagocytosis and absence of *in vivo* data on the roles of conformational epitopes of VTN in the binding to P47.

We showed that VTN inhibited the engulfment of SE36-beads in an anti-SE36 IgG-independent manner (Fig. 5b,c). It was reported that VTN inhibits interaction between the phagocytes and the apoptotic target cells. In this process, SmB domain and RGD motif of VTN inhibit efferocytosis through selective binding to integrins on macrophages and by binding to uPAR on apoptotic cells^26^. Although SE36 contains RGD motif (Fig. 2a), the relationship of this motif and engulfment remains unknown.

CD5 antigen-like (also known as SPα) and monocyte differentiation antigen CD14 had high ratio of the total spectrum counts between SE36 and Control columns by shotgun LC-MS/MS analysis (Supplementary Table 1; 26.00 and 11.00, respectively). These proteins are secreted in the serum and play an important role in the regulation of innate and adaptive immune systems^38,39^. Other serum proteins such as complements and apolipoproteins also had higher ratio than vitronectin. But the ELISA-based binding assay showed that these proteins did not bind directly to SE36 (Supplementary Fig. 3). With current results, binding can be partially attributed to interaction with VTN. Further studies on these interactions are definitely needed. Other hyperimmune sera are also best to be explored in the future.

It is reasonable to speculate that P47 fragment on the merozoite surface binds to VTN that further binds to many host factors. In fact, serum proteins such as thrombin, antithrombin III, and terminal complement complex were localized on the merozoite surface (Fig. 6), consistent with the list of SE36 affinity column binding proteins (Supplementary Table 1). These proteins, except antithrombin III, were removed in VTN-depleted serum (Supplementary Fig. 9b). Although, we could not completely remove antithrombin III, probably due to indirect binding to P47 fragment through thrombin^31^, VTN interaction with a plethora of binding partners has been implicated to contribute to immune evasion^40,41^ (for review, ref.^42^). Bacterial and fungal pathogens bind to VTN on their surface via a C-terminal heparin-binding domain (amino acids 352–374) avoiding the complement C5b-9 complex activation that can result to cell lysis^41^. Among them, in *Streptococcus pneumoniae* serotype 3 strains, a central core of factor H-binding inhibitor of complement (Hic) (amino acid 151–201), a member of the pneumococcal surface protein C (PspC) family, interacts with VTN preventing the formation of the terminal complement complex^40^. In *Rickettsia*, RC1281/Adr1 and RC1282/Adr2 contributed to evasion of complement-mediated killing by binding to VTN and factor H^43^. The central region of Hic (the predicted binding site of VTN in the pneumococcal Hic molecule)^40^ and the predicted surface exposed loops of the highly conserved outer membrane protein Adr2 in *Rickettsia*^43^ bear no structural similarity with the far end C-terminal 18 residues of peptide-15 using the BLAST algorithm at NCBI (https://blast.ncbi.nlm.nih.gov/Blast.cgi). Furthermore, although the impact of VTN glycosylation in SE36 binding needs to be clarified in future studies, oligosaccharide residues in VTN were also not required for binding of UspA2 and Hic in *Moraxella catarrhalis* and *Streptococcus pneumoniae*, respectively^40,44^. In *Plasmodium*, the role of naïve IgM both on the merozoite as well as the iRBC surface in host cell immune evasion^45,46^ and the role of complement inhibitors such as factor H and C1 esterase inhibitor bound to merozoite surface proteins have been reported^47,48^. Indeed, from the pathogen side, immune evasion is best accomplished not by a single host protein but likely is a multifunctional process. Thus, host factors surrounding the merozoite could function to camouflage the merozoite against host immunity.

Recently, Collins and colleagues constructed a parasite line where SERA5 was conditionally disreputable^49^. Since this parasite line could elucidate on the binding of VTN and its related proteins to SERA5, it is worth testing this parasite line in the future.

## Methods

### Ethics statement and serum preparation

The study used residual serum samples from participants in an earlier epidemiological study where ethical clearance for sampling and consent was obtained and approved by the Uganda National Council for Science and Technology^12^. Pooled serum with high anti-SE36 IgG titer were from healthy adults living in the malaria endemic area of Apac, Uganda. Analysis of serum samples from Japanese healthy volunteers was approved by the institutional review committee of the Research Institute for Microbial Diseases (RIMD), Osaka University (approval number: 22-3) and all experiments were performed in accordance with relevant approved guidelines and regulations. Informed consent was obtained from all participants. Hereinafter, pooled Ugandan high anti-SE36 IgG-titer serum and pooled Japanese naive human serum are referred to as “HTS” and “NHS”, respectively. All blood samples were unlinked and coded during blood collection, processed within a few hours after collection and the sera stored at −20 °C and −80 °C until analyses.

### Reagents and antibodies

Anti-SE36 rabbit and mouse sera were as previously described^15,50^. Anti-VTN monoclonal antibody (mAb) (clone: 8E6; MAB88917) and anti-VTN polyclonal antibody (pAb) (15833-1-AP) were purchased from Merck Millipore (Bedford, MA, USA) and Proteintech (Chicago, IL, USA), respectively. Anti-human serum albumin (HSA) pAb (A80-129P) was from Bethyl Laboratories (Montgomery, TX, USA), Anti-band 3 mAb (ab108414), anti-complement C7 mAb (ab126786), anti-complement C9 mAb (ab173302), anti-SC5b-9 complex pAb (ab55811), and isotype control (mouse IgG1) (ab81032) were from Abcam (Cambridge, UK). Anti-prothrombin pAb (24295-1-AP), anti-antithrombin III pAb (16414-1-AP), and isotype control (rabbit IgG) (30000-0-AP) were from Proteintech. Horseradish peroxidase (HRP)- and fluorescein-conjugated secondary antibodies (anti-mouse IgG-HRP (115-035-166), anti-rabbit IgG-HRP (711-035-152), anti-mouse IgG-FITC (515-095-062), and anti-rabbit IgG-Alex Flour594 (711-585-152)) were from Jackson ImmunoResearch Laboratories (West Grove, PA, USA).

### Recombinants and peptides

SE36 was expressed in *E. coli* using a codon optimized synthetic gene and purified as previously described^13^. In brief, SE36 without the serine repeats (encoding amino acid residues 17–192 and 226–382) was constructed from recombinant P47 (17–382 aa) of *P. falciparum* (Honduras-1) SERA5. For protein production, the plasmid, pET-SE36, was transformed into *E. coli* BL21 (DE3) pLysS and expression of the recombinant protein was induced with 50 µg/mL Isopropyl-β-D-thiogalactoside. SE36 protein fraction was purified initially by ion exchange column (DEAE Sepharose FF: GE Healthcare, Little Chalfont, UK) and the flow-through subjected to 25% ammonium sulfate fractionation. Succeeding steps in SE36 protein purification involves Sephacryl S-300 HR (GE Healthcare), Octyl Sepharose 4FF (GE Healthcare) and Superdex 200 pg (GE Healthcare) gel filtration under denaturing conditions. Protein fractions of SE36 were pooled, refolded by gradual dialysis against phosphate-buffered saline (PBS) and passed through 0.2 µm filter. Purified SE36 protein was stored at ≤−20 °C until use. Fifteen synthetic peptides of 40–42 residues covering the whole sequence of SE36 were synthesized as previously described^15^. Purified VTN was purchased from Promega (Madison, WI, USA), and VTN recombinants, Recombinant Human Vitronectin (62–398 aa) Protein (Cat #: HRP-0323) and Recombinant Human Vitronectin Protein, CF (Cat #: 2308-VN), were obtained from LD Biopharma (San Diego, CA, USA) and R&D systems (Boston, MA, USA), respectively. Truncated recombinants of SE36 and VTN were prepared as described below. All recombinants and peptides are schematically summarized in Figs 1e and 2a.

### Affinity purification of SE36-binding proteins

Affinity purification was performed using the AminoLink Plus Immobilization Kit (Pierce, Rockford, IL, USA) according to the manufacturer’s instructions. Briefly, NHS was diluted (1:10) in PBS (pH 7.4) containing complete proteinase inhibitor cocktail (Roche Biochemicals, Mannheim, Germany) and applied to the SE36-immobilized and non-SE36-immobilized (control) columns. The columns were subjected to end-over-end rotation for 1 h at room temperature. The columns were extensively washed with PBS containing the proteinase inhibitor cocktail followed by elution of bound proteins with 2 mL 0.1 M glycine-HCl buffer (pH 2.7). The binding proteins were eluted four times. Elutes were neutralized by 50 μL 1M Tris-HCl (pH 9.0) and subjected to sodium dodecyl sulphate-polyacrylamide gel electrophoresis (SDS-PAGE) and the bands were visualized by silver staining using SilverQuest (Thermo Fisher Scientific, Waltham, MA, USA) (Supplementary Fig. 2).

### Shotgun liquid chromatography–tandem mass spectrometry (LC-MS/MS)

Each eluate from Elution-2 was concentrated using a 10 kDa MWCO Amicon Ultra-4 centrifugal filter unit (Merck Millipore) and then subjected to SDS-PAGE. The samples were digested in-gel with trypsin, and analyzed by nanocapillary reversed-phase LC–MS/MS using the Magic C18 reverse phase column on a nano LC system (Thermo Fisher Scientific) coupled to a quadrupole time-of-flight mass spectrometer (QTOF Ultima, Waters, Milford, MA, USA). Direct injection data-dependent acquisition was performed using one MS channel for every three MS/MS channels and dynamic exclusion for selected ions. Proteins were identified by database search using the Mascot Server (Matrix Science, Boston, MA, USA).

### Cloning, expression, and purification of recombinant SE36-1 to -4

*Eco*RV and *Not*I sites were introduced at the end of the forward and reverse primers, respectively (restriction enzyme sites are underlined): SE36-1, SE36-1/Fwd (5′-GAGAGAGAGATATCTCATGAAAAACGTGATCAAATG-3′) and SE36-1/Rev (5′-GAGAGAGAGCGGCCGCTTAGGTATTTGTTTTTCAGAAG-3′); SE36-2, SE36-2/Fwd (5′-GAGAGAGAGATATCTCATGGAAAAACAAGATACCATTCAG-3′) and SE36-2/Rev (5′-GAGAGAGAGCGGCCGCTTAACTCGAGTCTGAAATCGGTTC-3′); SE36-3, SE36-3/Fwd (5′-GAGAGAGAGATATCTCATGTCGAGTGAAAGTCTTCCGGC-3′) and SE36-3/Rev (5′-GAGAGAGAGCGGCCGCTTATTTTTCCGGAATGTCTGAGC-3′); and SE36-4, SE36-4/Fwd (5′-GAGAGAGAGATATCTCATGGAAAAATGTGATACCCTGGC-3′) and SE36-4/Rev (5′-GAGAGAGAGCGGCCGCTTACTCCGCTTTGATCTCCTTG-3′). SE36-1 to -4 were generated according to the sequence of the codon-optimized SE36 gene^13^. The amplified fragments were restriction-digested, ligated in-frame into the *Eco*RV and *Not*I sites of the pEU-E01-His-TEV-MCS-N2 expression vector for the wheat germ cell-free translation system (CellFree Sciences, Matsuyama, Japan). Recombinants were expressed using the WEPRO7240H Expression Kit (CellFree Sciences) and purified by Ni-sepharose 6 Fast Flow (GE Healthcare).

### Cloning, expression and purification of recombinant VTN-1 to -4 and -2-1 to -2-3

*Eco*RI and *Not*I sites were introduced at the end of the forward and reverse primers, respectively (restriction enzyme sites are underlined): VTN-1, VTN-F1(EcoRI) (5′-GCGAATTCGACCAAGAGTCATGCAAGGG-3′) and VTN-R1(NotI) (5′-GCGCGGCCGCCAGCTCCTCCTCTGCTGGGG-3′); VTN-2, VTN-F2(EcoRI) (5′-GCGAATTCGAGGAGCTGTGCAGTGGGAAG-3′) and VTN-R2(NotI) (5′-GCGCGGCCGCCATCATGGCAAAGTGTTCAAAC-3′); VTN-3, VTN-F3(EcoRI) (5′-GCGAATTCGCCATGATGCAGCGGGACAG-3′) and VTN-R3(NotI) (5′-GCGCGGCCGCGCGGGATGGCCGGCGGGAG-3′); VTN-4, VTN-F4(EcoRI) (5′-GCGAATTCGCCATGTGGCTGTCCTTGTTC-3′) and VTN-R4(NotI) (5′-GCGCGGCCGCCAGATGGCCAGGAGCTGGGC-3′); VTN-2-1, VTN-F2(EcoRI) and VTN-R2-1(NotI) (5′-GCGCGGCCGCGGGGCCCTCGATGCCCCAGA-3′); VTN-2-2, VTN-F2-2(EcoRI) (5′-GCGAATTCTGGGGCATCGAGGGCCCCAT-3′) and VTN-R2-2(NotI) (5′-GCGCGGCCGCGTTGTCCGGGATGCCATCGA-3′); and VTN-2-3, VTN-F2-3(EcoRI) (5′-GCGAATTCGATGGCATCCCGGACAACGT-3′) and VTN-R2 (NotI). The amplified fragments were digested and ligated in-frame into the *Eco*RI and *Not*I sites of the pPICZα A expression vector for *Pichia pastoris* Expression System (Thermo Fisher Scientific). All constructs were linearized by *Pme*I digestion and then transfected to GS115 strain using GenePulser Xcell (Bio-Rad, Hercules, CA, USA). Recombinants were expressed and purified by His GraviTrap (GE Healthcare) according to the manufacturer's protocol.

### ELISA-based direct binding assay

SE36, SE36-1 to -4 recombinants, purified VTN, VTN recombinants (1 μg/mL), or SE36 peptides (0.3 μM) were suspended in 100 μL carbonate/bi-carbonate buffer (pH 9.6) and coated onto flat-bottom 96-well MaxiSorp NUNC-Immuno plates (Nunc, Roskilde, Denmark) overnight at 4 °C. The plates were washed with PBS-0.05% Tween 20 and blocked for 1 h at room temperature with PBS-0.05% Tween 20 containing 5% skim milk. After washing, the wells were incubated with human serum (diluted 1:2000), purified or recombinant VTN (2 μg/mL), or SE36 (2 μg/mL) in PBS-0.05% Tween 20 containing 5% skim milk for 2 h at room temperature. Anti-SE36 mouse serum (diluted 1:1000), anti-VTN mAb (diluted 1:2000), or anti-VTN pAb (diluted 1:5000) was added after the washing steps prior to another incubation for 2 h at room temperature. This was followed by incubation with HRP-conjugated anti-mouse pAb (diluted 1:10000) or anti-rabbit pAb (diluted 1:10000) for 2 h at room temperature. Color was developed using TMB microwell peroxidase substrate (KPL, Gaithersburg, MD, USA). Reactions were stopped by addition of 1 M sulfuric acid. Absorbance was measured at 450/540 nm.

### Surface plasmon resonance (SPR)-Biacore

SPR experiments were performed on a Biacore T200 instrument using a Series S Sensor Chip SA (GE Healthcare). The biotinylated SE36 was immobilized at a level 200 RU via streptavidin on the biosensor. On-rate (*k*_a_) and off-rate (*k*_d_) profiles were measured using flow rates of 20 μL/min at 25 °C in filtered PBS containing 0.05% Tween 20 (PBS-T) running buffer including purified VTN [20 μg/mL (~285.7 nM), 10 μg/mL (~142.9 nM), 5 μg/mL (~71.4 nM), 2.5 μg/mL (35.7 nM), and 1.25 μg/mL (~17.9 nM)]. The data were fitted to 1:1 binding and bivalent analyte models using numerical integration algorithms available in BIAevaluation software.

### Preparation of RBC and infected RBC (iRBC) lysates

*P. falciparum* strain 3D7 was cultured in RPMI 1640 medium supplemented with 0.5 g/L L-glutamine, 5.96 g/L HEPES, 2 g/L NaHCO_3_, 50 mg/L hypoxanthine, 10 mg/L gentamicin, and 10% heat-inactivated human serum or 5 mg/mL AlbuMAX-I, and RBCs at 3% hematocrit in an atmosphere of 5% CO_2_, 5% O_2_, and 90% N_2_ at 37 °C as previously described^51^. Ring-stage iRBCs were collected by sorbitol synchronization^52^. Briefly, culture contents were collected by centrifugation. The supernatant was discarded and the pelleted cells were suspended in 5 volumes of 5% D-sorbitol and allowed to stand for 10 min. The cells were washed twice with RPMI 1640 medium to remove sorbitol and adjusted to ~1.5% parasitemia at 3% hematocrit. The collected ring-stage iRBCs were cultured for ~18 hours and then enriched by 58.5% Percoll density centrifugation as described by Tosta *et al*.^53^. RBC membrane was lysed using 0.075% saponin. The lysates were prepared for western blotting.

### Western blotting

Protein concentration in each sample was accurately estimated using NanoDrop 1000 (Thermo Fisher Scientific). Samples were resuspended in the SDS-containing sample buffer supplemented with a reducing reagent, and resolved on NuPAGE 4–12% Bis-Tris gels using 1 × NuPAGE MES SDS Running Buffer (Thermo Fisher Scientific). The separated proteins were transferred onto polyvinylidene fluoride membranes using iBlot (Thermo Fisher Scientific). Membranes were incubated in the blocking buffer [PBS containing 0.05% (v/v) Tween 20 (PBS-T) with 5% (w/v) skim milk] for 30 min. Next, they were placed in the blocking buffer containing primary antibodies or antiserum (dilution rate of each primary antibody or antiserum was indicated in Figure legend) for 1 h. After thrice washing with PBS-T, the blots were incubated in blocking buffer containing the respective secondary antibody (1:10000; anti-mouse IgG-HRP, anti-rabbit IgG-HRP, or anti-goat IgG-HRP) for 1 h. Membranes were washed with PBS-T, soaked in the TMB Microwell Peroxidase Substrate System (KPL, Gaithersburg, MD, USA), and analyzed using LAS 4000 (GE Healthcare).

### Imaging

Parasites were fixed on slides with 100% methanol, permeabilized with 0.1% Triton X-100, and blocked with 1% bovine serum albumin (BSA). Slides were then incubated with primary antibodies for 1.5 h at room temperature and then washed thrice to remove unbound antibodies. All primary antibodies were diluted to 1:200. Slides were then incubated with secondary antibody [FITC-conjugated anti-mouse pAb (diluted 1:100) or Alexa Fluor 594- conjugated anti-rabbit pAb (diluted 1:200)] for 1.5 h and mounted in Vectashield mounting medium with DAPI (Vector Laboratories, CA, USA). Images were captured using the confocal laser-scanning microscope, FluoView FV10i (Olympus, Tokyo, Japan), the DeltaVision deconvolution microscopy system (GE healthcare), and KEYENCE fluorescence microscope BZ-X710 (Keyence, Osaka, Japan).

### Phagocytosis Assay

SE36 was biotinylated by ECL Protein Biotinylation Module (GE healthcare) and then the biotinylated SE36 was coated on 1 μm yellow-green fluorescent (505/515 nm) neutravidin beads (Thermo Fisher Scientific). IgG was purified using the Melon Gel IgG Purification kit (Thermo Fisher Scientific) and re-suspended in PBS. The VTN-depleted sera were prepared by anti-VTN IgG-immobilized column using AminoLink Plus Immobilization Kit according to the manufacturer's instructions (Thermo Fisher Scientific). Phagocytosis assays were performed as previously described in ref.^54^, with minor adjustments. In brief, 5 × 10^4^ THP-1 cells were added to each well in a final volume of 100 μL, and the plate was incubated overnight under standard tissue culture conditions (37 °C, 5% CO_2_). After incubation, 5 × 10^5^ beads were placed in each well of flat-bottom 96-well plates and incubated for 6 h. Cells were washed in ice-cold PBS by centrifugation at 500 *g* for 5 min at 4 °C. In each experiment, at least 10,000 events were recorded using FACSCalibur flow cytometer (FCM) and analysed using CellQuest Pro Version 5.2.1 software (BD Biosciences, Franklin, NJ, USA). The phagocytosis index (PI) (%) was calculated from the ratio of median fluorescent intensity (MFI) of THP-1 cells that engulfed tested beads/MFI of THP-1 cells that engulfed SE36-beads × 100.

### Statistical analysis

Standard deviation (SD) was calculated by Microsoft Excel software (Microsoft, Roselle, IL, USA). Comparisons of VTN-binding in sera (NHS and HTS) were analysed by a two-tailed non-parametric Mann-Whitney U test. The significance limit was set at *p* values of <0.05. Calculations were performed using the Graphpad Prism 5 (Graphpad Prism Software, San Diego, CA, USA).

### Data Availability

The datasets generated during and/or analysed during the current study are available from the corresponding author on reasonable request.

## Electronic supplementary material

Supplementary Information

Supplementary Movie 1
