# Supplementary material for: Molecular Camouflage of Plasmodium falciparum Merozoites by Binding of Host Vitronectin to P47 Fragment of SERA5
Source: Sci Rep. 2018 Mar 22;8:5052. doi: 10.1038/s41598-018-23194-9 (PMC5864917; doi:10.1038/s41598-018-23194-9)
Supplement: Supplementary file 1 — Supplementary Information [file 41598_2018_23194_MOESM1_ESM.pdf]

Supplementary Information

Molecular Camouflage of *Plasmodium falciparum* Merozoites by Binding of Host Vitronectin to P47 Fragment of SERA5

Takahiro Tougan[1], Jyotheeswara R. Edula[1], Eizo Takashima[2], Masayuki Morita[2], Miki Shinohara[3], Akira Shinohara[4], Takafumi Tsuboi[2] & Toshihiro Horii[1,*]

[1]Department of Molecular Protozoology, Research Institute for Microbial Diseases, Osaka University, 3-1 Yamadaoka, Suita, Osaka 565-0871, Japan

[2]Division of Malaria Research, Proteo-Science Center, Ehime University, 3 Bunkyo-cho, Matsuyama, Ehime 790-8577, Japan

[3]Department of Advanced Bioscience, Faculty of Agriculture, Kindai University, 3327-204 Nakamachi, Nara, Nara 631-8505, Japan

[4]Department of Integrated Protein Functions, Institute for Protein Research, Osaka University, 3-2 Yamadaoka, Suita, Osaka 565-0871, Japan

[*]Corresponding author. Tel.: +81-(0)6-6879-8280; fax: +81-(0)6-6879-8281

Email address: horii@biken.osaka-u.ac.jp (T. Horii)

Table of contents

Supplementary Methods

Supplementary Methods references

Figure legends

1. **Supplementary Figure 1.** Demonstration of the binding of SE36 against serum protein(s), Related to Figure 1
2. **Supplementary Figure 2.** Elution of SE36-binding proteins using SE36-immobilized column, Related to Figure 1
3. **Supplementary Figure 3.** Binding assay of serum proteins to SE36, Related to Figure 1
4. **Supplementary Figure 4.** Purity of SE36-1 to -4 recombinants, Related to Figure 2
5. **Supplementary Figure 5.** Confirmation of binding of SE36 to the latex beads, Related to Figure 5
6. **Supplementary Figure 6.** Engulfment of thrombin-coated beads by THP-1 cells, Related to Figure 5
7. **Supplementary Figure 7.** Inhibition of engulfment of SE36-beads by cytochalasin D (CytD), Related to Figure 5
8. **Supplementary Figure 8.** Binding of SE36 to THP-1 cells with or without VTN, Related to Figure 5
9. **Supplementary Figure 9.** Confirmation of depletion of VTN from sera, Related to Figure 5
10. **Supplementary Figure 10.** Confirmation of binding of SE36 and VTN to the latex beads, Related to Figure 5
11. **Supplementary Figure 11.** Confirmation of presence of VTN in both of NHS and HTS, Related to Figure 5
12. **Supplementary Figure 12**. Full Blots to Figure 3c
13. **Supplementary Figure 13.** Full Blots to Figure 5a(i)
14. **Supplementary Figure 14.** Full Blots to Supplementary Figure 5a
15. **Supplementary Figure 15.** Full Blots to Supplementary Figure 6a
16. **Supplementary Figure 16.** Full Blots to Supplementary Figure 8a
17. **Supplementary Figure 17.** Full Blots to Supplementary Figure 8b

18. **Supplementary Figure 18.** Full Blots to Supplementary Figure 9b
19. **Supplementary Figure 19.** Full Blots to Supplementary Figure 10a
20. **Supplementary Figure 20.** Full Blots to Supplementary Figure 10b
21. **Supplementary Figure 21.** Full Blots to Supplementary Figure 11
22. **Supplementary Table 1.** Identification of SE36 binding proteins by shotgun LC-MS/MS analysis, Related to Figure 1
23. **Supplementary Table 2.** Molecules and antibodies used for ELISA-based binding assay, Related to Supplementary Figure 3
24. **Supplementary Movie 1.**

Supplementary Methods

Preparation of VTN-depleted serum. The depletion of VTN was performed using the AminoLink Plus Immobilization Kit (Thermo Fisher Scientific) according to the manufacturer's instructions. Briefly, NHS or HTS was diluted (1:10) in PBS (pH 7.4) and applied to the anti-VTN antibody-immobilized column. The column was subjected to end-over-end rotation for 1 h at room temperature. Flow-through were collected and concentrated using a 10 kDa MWCO Amicon Ultr-4 centrifugal filter unit (Merck Millipore). The depletion was confirmed by CBB staining and western blotting probed with anti-VTN pAb (diluted 1:5000).

Preparation of thrombin-coated beads. Thrombin from human plasma (Cat #: T1063) was purchased from Sigma-Aldrich. Thrombin was biotinylated by ECL Protein Biotinylation Module (GE healthcare). The biotinylated thrombin was coated on 1 μm yellow-green fluorescent (505/515 nm) neutravidin beads (Thermo Fisher Scientific). The binding of thrombin to the beads was confirmed by anti-thrombin pAb (24295-1-AP) (diluted 1:5000, Proteintech).

Inhibition of phagocytosis by cytochalasin D. To inhibit phagocytosis, THP-1 cells

were preincubated with 5 μM cytochalasin D (CytD; Wako, Osaka, Japan), an actin polymerization inhibitor, for 1 h at 37°C prior to the addition of SE36-beads as previously described[55]. The phagocytosis assay was performed as mentioned in Methods.

Competition assay of VTN against SE36-binding to THP-1 cells. For confirmation of the bindings of SE36 and VTN to THP-1 cells, sequentially diluted SE36 or VTN (15, 5, 1.5, and 0.5 μg/mL) were added to 1×10^5 THP-1 cells with a final volume of 100 μL in each well and incubated for 3 h under standard tissue culture conditions (37°C, 5% CO_2). After incubation, cells were washed with ice-cold PBS by centrifugation at 500 *g* for 5 min at 4°C. The binding of SE36 and VTN were confirmed by anti-SE36 mouse serum (diluted 1:1000) and anti-VTN pAb (15833-1-AP) (diluted 1:5000, Proteintech), respectively. For competition assay, 15 μg/mL SE36 and sequentially diluted SE36 or VTN (15, 5, 1.5, and 0.5 μg/mL) were added to 1×10^5 THP-1 cells. The bindings of SE36 and VTN was confirmed as above.

Holotomography. In order to measure three-dimensional (3-D) refractive index (RI) distributions of cells, we utilized commercialized holotomography setup (HT-1S, Tomocube, Inc., Daejeon, South Korea). Holotomography or optical diffraction

tomography is a laser holographic technique which measures multiple two-dimensional holograms of a sample at various illumination angles, from which a 3-D RI tomogram of the sample is reconstructed[56,57]. The optical system was based on a Mach-Zehnder interferometric microscope equipped with a DMD[58]. A laser beam from a diode-pumped solid-state laser (l = 532 nm, 10 mW) was divided into two arms. One beam was used as a reference beam, and the other beam illuminated a sample with various incident angles ranging from -53° to 53°. In order to control the angle of incident illumination, a digital micromirror device (DMD, DLP6500FYE, Texas Instruments Inc., Dallas, Texas, USA) was implemented. By projecting a Lee hologram pattern on a DMD, the angle of the diffracted light from the DMD was precisely controlled by applying corresponding hologram patterns. The beam diffracted from the sample was collected by using a 60× objective lens, and then was interfered with the reference beam, generating spatially modulated holograms. The holograms of a sample were recorded with a high-speed image sensor (CMOS camera, FL3-U3-13Y3M-C, FLIR Systems, Inc., Wilsonville, OR, USA) with a frame rate of 300 Hz.

Supplementary Methods-only references

55. Chan, C. L., Rénia, L., & Tan, K. S. A simplified, sensitive phagocytic assay for

malaria cultures facilitated by flow cytometry of differentially-stained cell populations. *PLoS One* **7**, e38523 (2012).

56. Wolf, E. Three-dimensional structure determination of semi-transparent objects from holographic data. *Optics Communications* **1**, 153-156 (1969).

57. Lee, K. et al. Quantitative phase imaging techniques for the study of cell pathophysiology: from principles to applications. *Sensors (Basel)* **13**, 4170-4191 (2013).

58. Shin, S. et al. Optical diffraction tomography using a digital micromirror device for stable measurements of 4-D refractive index tomography of cells. *Proc. SPIE*, 971814 (2016).

Supplementary Figure legends

Supplementary Figure 1. Demonstration of the binding of SE36 against serum protein(s), Related to Figure 1. Reactivity of SE36 against serum protein(s). Naïve human serum (NHS) was adsorbed on microtiter plate at indicated dilutions and SE36

was added at various concentrations. After incubation, SE36 was detected by anti-SE36 mouse serum (diluted 1:1000).

Supplementary Figure 2. Elution of SE36-binding proteins using SE36-immobilized column, Related to Figure 1. Visualization of the eluted proteins by sodium dodecyl sulphate-polyacrylamide gel electrophoresis (SDS-PAGE) and silver staining. “SE36” and “Control” indicate samples eluted from SE36- and control columns, respectively. The control column is simply without SE36 protein and is referred to as “non-SE36-immobilized” (control) column. The elution step was repeated four times, indicated as Elution-1 to -4.

Supplementary Figure 3. Binding assay of serum proteins to SE36, Related to Figure 1. a, (i) Reactivity of purified VTN, C5, C7, C8, C9, and fH against SE36. (ii) Reactivity of purified VTN, ApoA1, HDL, and LDL against SE36. (iii) Reactivity of VTN, THRB, CLU, and FINC against SE36. SE36 was adsorbed to microtiter plate and the purified molecules were added at various dilutions. Each molecule was detected by each corresponding antibody. **b**, (i) Reactivity of anti-VTN mAb, anti-C5 pAb, anti-C7 mAb, anti-C8 mAb, anti-C9 mAb, and anti-fH mAb to each corresponding antibody. (ii)

Reactivity of anti-VTN mAb, anti-ApoA1 pAb, anti-HDL pAb, and anti-LDL pAb to each corresponding antibody. (iii) Reactivity of anti-VTN mAb, anti-THRB pAb, anti-CLU mAb, and anti-FINC mAb to each corresponding antibody. Each molecule was adsorbed to microtiter plate at the indicated dilutions. **c**, Reactivity of VTN, CD5L, and CD14 in NHS against SE36. SE36 was adsorbed to microtiter plate and NHS was added at the indicated dilutions. **d**, Reactivity of anti-VTN mAb, anti-CD5L pAb, and anti-CD14 mAb to corresponding proteins in NHS. At the different dilutions used for NHS we could not detect CD5L and CD14. Thus, although both serum proteins could be detected in LC-MS/MS, at several concentrations we could not demonstrate direct binding to SE36. NHS was adsorbed to microtiter plate at the indicated dilutions. Used proteins and antibodies are listed in Supplementary Table 2.

Supplementary Figure 4. Purity of SE36-1 to -4 recombinants, Related to Figure 2. SDS-PAGE and Coomassie Brilliant Blue (CBB) staining of each elute. E1 and E2 represent the first and second elutes from the Ni column used for purification via His tags. Two micrograms of protein samples were loaded. Arrows indicate the target recombinants.

Supplementary Figure 5. Confirmation of binding of SE36 to the latex beads,

Related to Figure 5. a, Western blotting of SE36 bound to beads. SE36 was detected by anti-SE36 mouse serum (diluted 1:1000). Arrow indicates the target protein, SE36. **b**, Representative IFA images of the latex beads (green) and SE36 (red) probed with anti-SE36 mouse serum. Scale bar, 5 μm.

Supplementary Figure 6. Engulfment of thrombin-coated beads by THP-1 cells, Related to Figure 5. a, Western blotting of thrombin bound to beads. Thrombin was detected by anti-prothrombin pAb (diluted 1:2000). Arrow indicates the target protein, thrombin. **b**, Representative flow cytometry (FCM) histogram of the engulfment of thrombin-beads by THP-1 cells. Numbers in parentheses are the phagocytosis index (PI) (%) for reference.

Supplementary Figure 7. Inhibition of engulfment of SE36-beads by cytochalasin D (CytD), Related to Figure 5. a, Representative FCM histogram of the engulfment of SE36-beads by THP-1 cells with or without CytD. **b**, Representative FCM histogram of the engulfment of beads by THP-1 cells with or without CytD. Numbers in parentheses indicate PI (%).

Supplementary Figure 8. Binding of SE36 to THP-1 cells with or without VTN, Related to Figure 5. a, Western blotting of SE36 and VTN bound to THP-1 cells. (i) Detection of SE36 bound to THP-1 cells. (ii) Detection of VTN bound to THP-1 cells. **b**, Competitive binding of SE36 and VTN to THP-1 cells. β-actin was used as loading control. Approximately 10 μg of cell lysate was run in an SDS-PAGE gel and probed with anti-SE36 mouse serum (diluted 1:1000), anti-VTN pAb (diluted 1:2000), and anti-β-actin (diluted 1:4000). Arrows indicate the target proteins.

Supplementary Figure 9. Confirmation of depletion of VTN from sera, Related to Figure 5. a, CBB staining shows sera, NHS and HTS, before and after the depletion of VTN. NHS or HTS was applied to anti-VTN pAb-immobilized column to deplete VTN. Approximately 3 μL of 1:30 diluted serum was run in an SDS-PAGE gel and stained with CBB. **b**, Western blotting of VTN in sera before and after the depletion of VTN. “Serum” indicates NHS or HTS. “VTN-depl” means VTN-depleted serum. Approximately 3 μL of 1:100 diluted serum was run in an SDS-PAGE gel and probed with each indicated antibody (all antibodies were used at a dilution of 1:2000). Arrows indicate the target proteins.

Supplementary Figure 10. Confirmation of binding of SE36 and VTN to the latex beads, Related to Figure 5. a, Western blotting of VTN and SE36 on SE36-beads treated with normal or VTN-depleted sera. **b**, Western blotting of VTN on SE36-beads treated with VTN-depleted NHS, VTN-depleted NHS with purified VTN or NHS. Proteins bound to the beads were detected by anti-SE36 mouse serum (diluted 1:1000) and anti-VTN pAb (diluted 1:2000). “VTN-depl” means VTN-depleted serum. Arrows indicate the target proteins.

Supplementary Figure 11. Confirmation of presence of VTN in both of NHS and HTS, Related to Figure 5. Western blotting of serum VTN in NHS and HTS. Serum albumin was used as a loading control. Approximately 3 μL of 1:100 diluted serum was run in an SDS-PAGE gel and probed with anti-VTN pAb (diluted 1:2000) and anti-HSA pAb (diluted 1:2000). Arrows indicate the target proteins.

Supplementary Figure 12. Full Blots to Figure 3c

Supplementary Figure 13. Full Blots to Figure 5a(i)

Supplementary Figure 14. Full Blots to Supplementary Figure 5a

Supplementary Figure 15. Full Blots to Supplementary Figure 6a

Supplementary Figure 16. Full Blots to Supplementary Figure 8a

Supplementary Figure 17. Full Blots to Supplementary Figure 8b

Supplementary Figure 18. Full Blots to Supplementary Figure 9b

Supplementary Figure 19. Full Blots to Supplementary Figure 10a

Supplementary Figure 20. Full Blots to Supplementary Figure 10b

Supplementary Figure 21. Full Blots to Supplementary Figure 11

Supplementary Table 1. Identification of SE36 binding proteins by shotgun LC-MS/MS analysis, Related to Figure 1.

Supplementary Table 2. Molecules and antibodies used for ELISA-based binding assay, Related to Supplementary Figure 3

Supplementary Movie 1. Confirmation of the engulfment of SE36-beads by THP-1 cell, Related to Figure 6. Representative image of holotomography shows THP-1 cell (purple) and SE36-beads (green). The 3-D refractive index distributions of the THP cell and SE36-beads are measured with a tomogram acquisition rate of 300 Hz. The theoretical optical lateral and axial resolution of the present method were calculated as 166 nm and 1.00 mm. Vertical bar indicates the relative refractive index distribution.

Supplementary Figure 1. Demonstration of the binding of SE36 against serum protein(s), Related to Figure 1

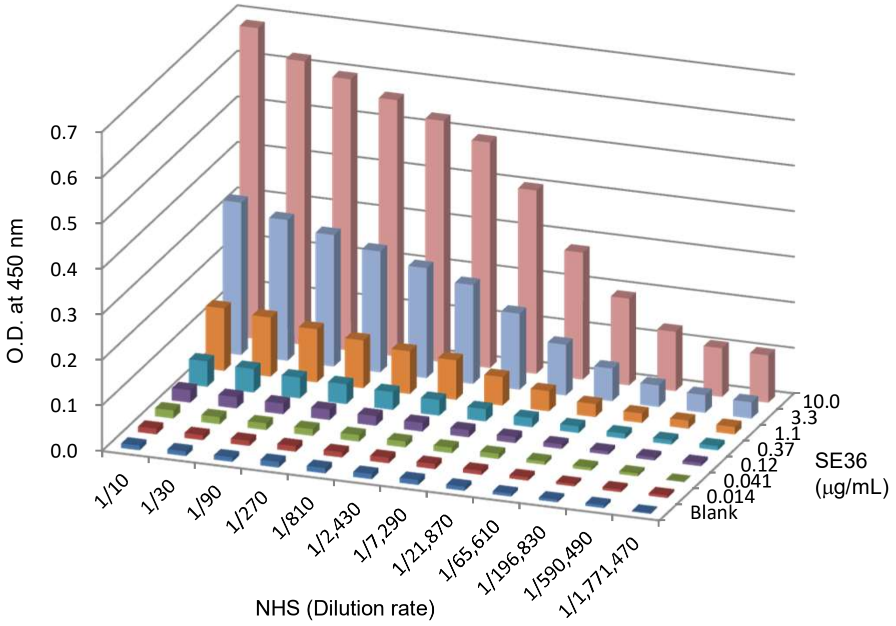

Supplementary Figure 2. Elution of SE36-binding proteins using SE36-immobilized column, Related to Figure 1

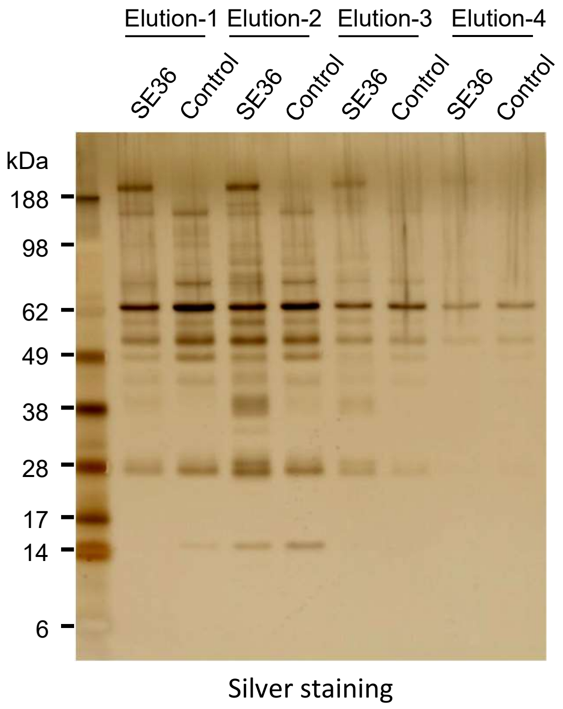

Supplementary Figure 3. Binding assay of serum proteins to SE36, Related to Figure 1

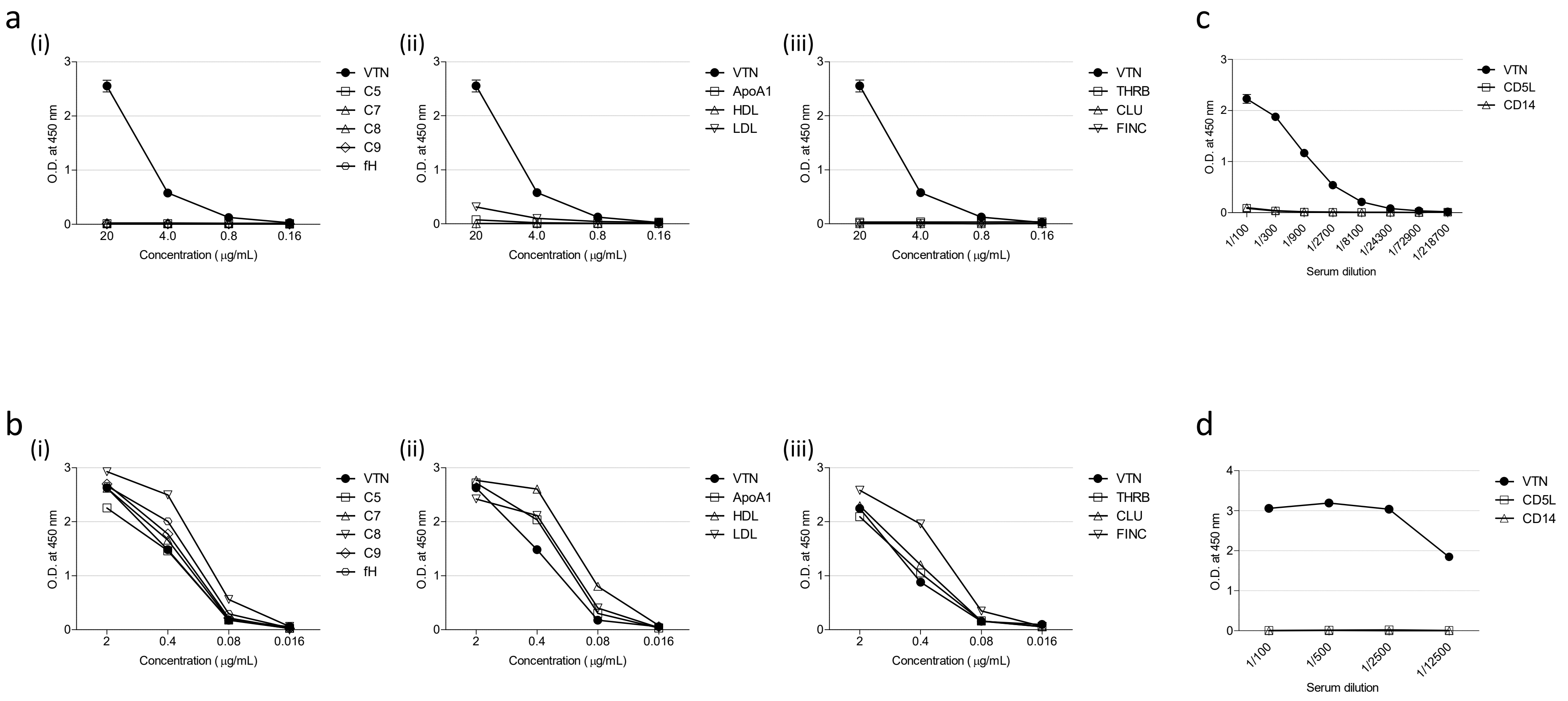

Supplementary Figure 4. Purity of SE36-1 to -4 recombinants, Related to Figure 2

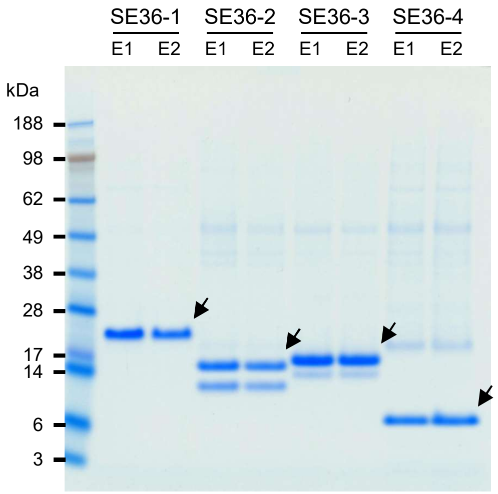

CBB staining

Supplementary Figure 5. Confirmation of binding of SE36 to the latex beads, Related to Figure 5

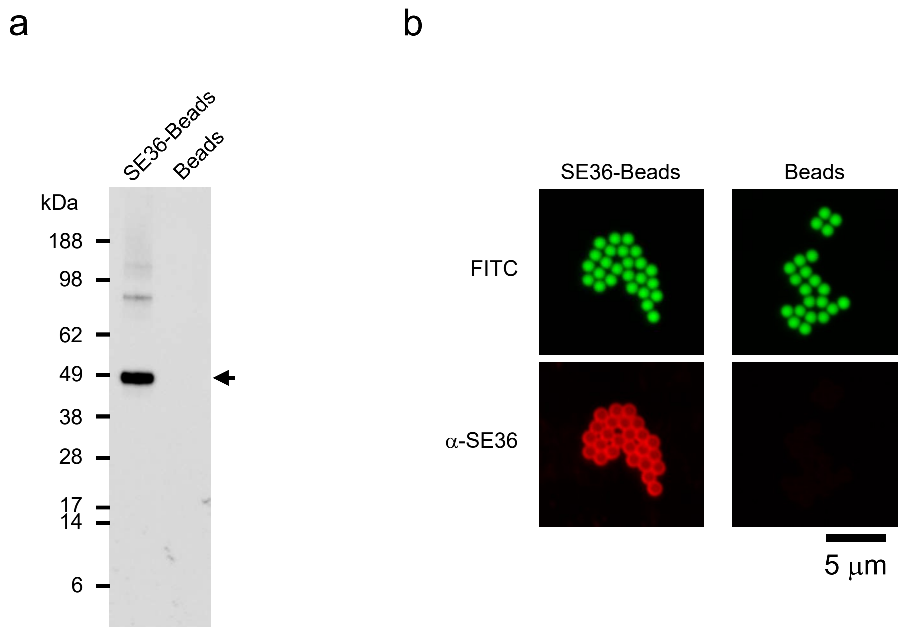

Supplementary Figure 6. Engulfment of thrombin-coated beads by THP-1 cells, Related to Figure 5

a

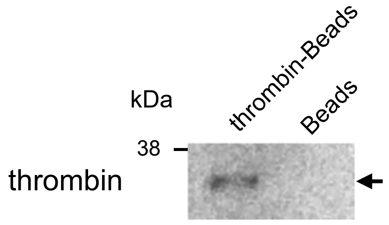

b

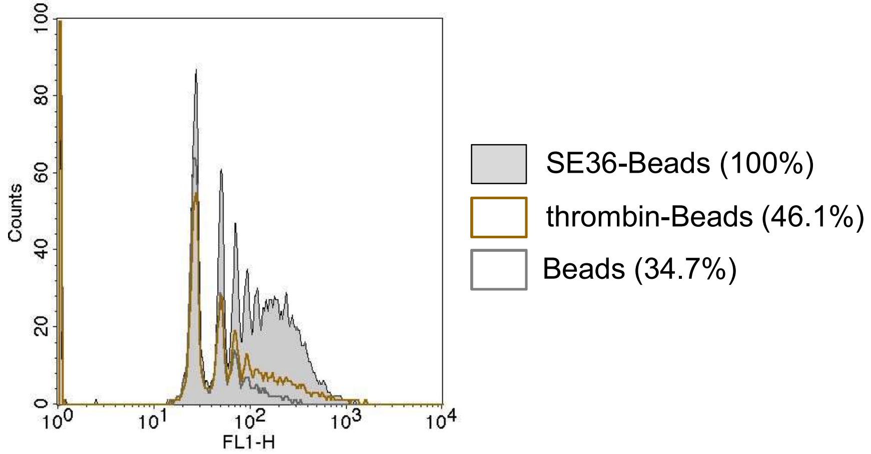

Supplementary Figure 7. Inhibition of engulfment of SE36-beads by cytochalasin D (CytD), Related to Figure 5

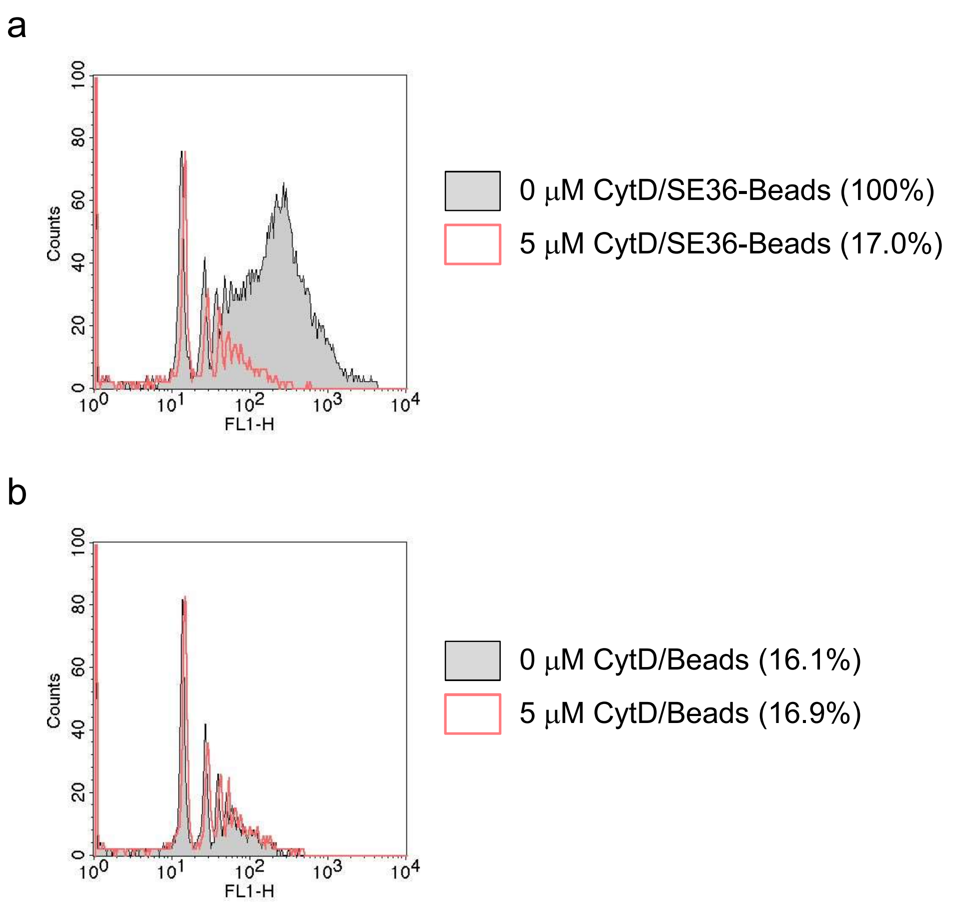

Supplementary Figure 8. Binding of SE36 to THP-1 cells with or without VTN, Related to Figure 5

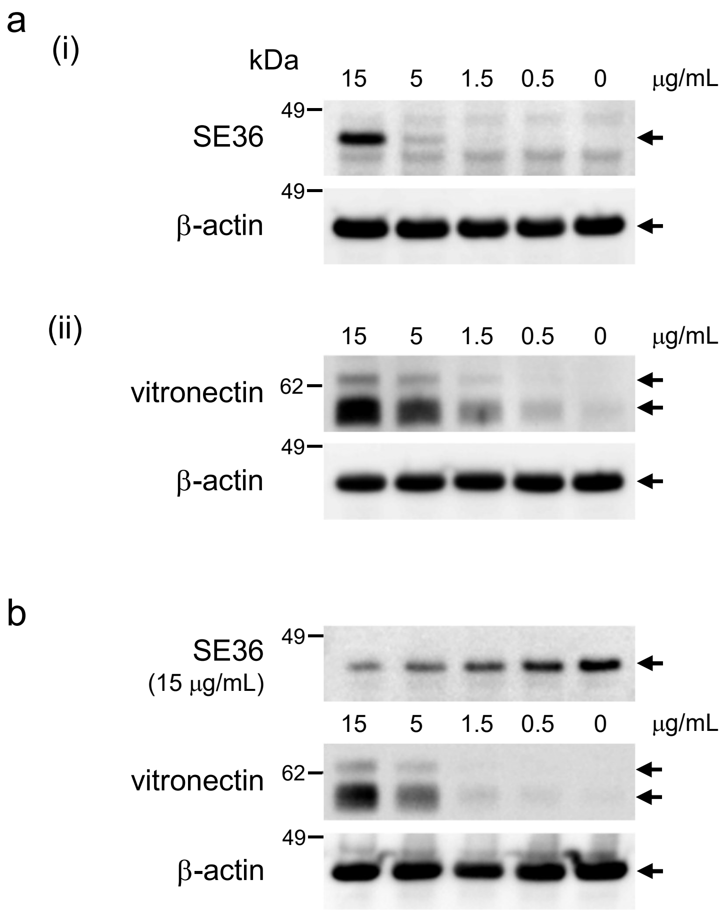

Supplementary Figure 9. Confirmation of depletion of VTN from sera, Related to Figure 5

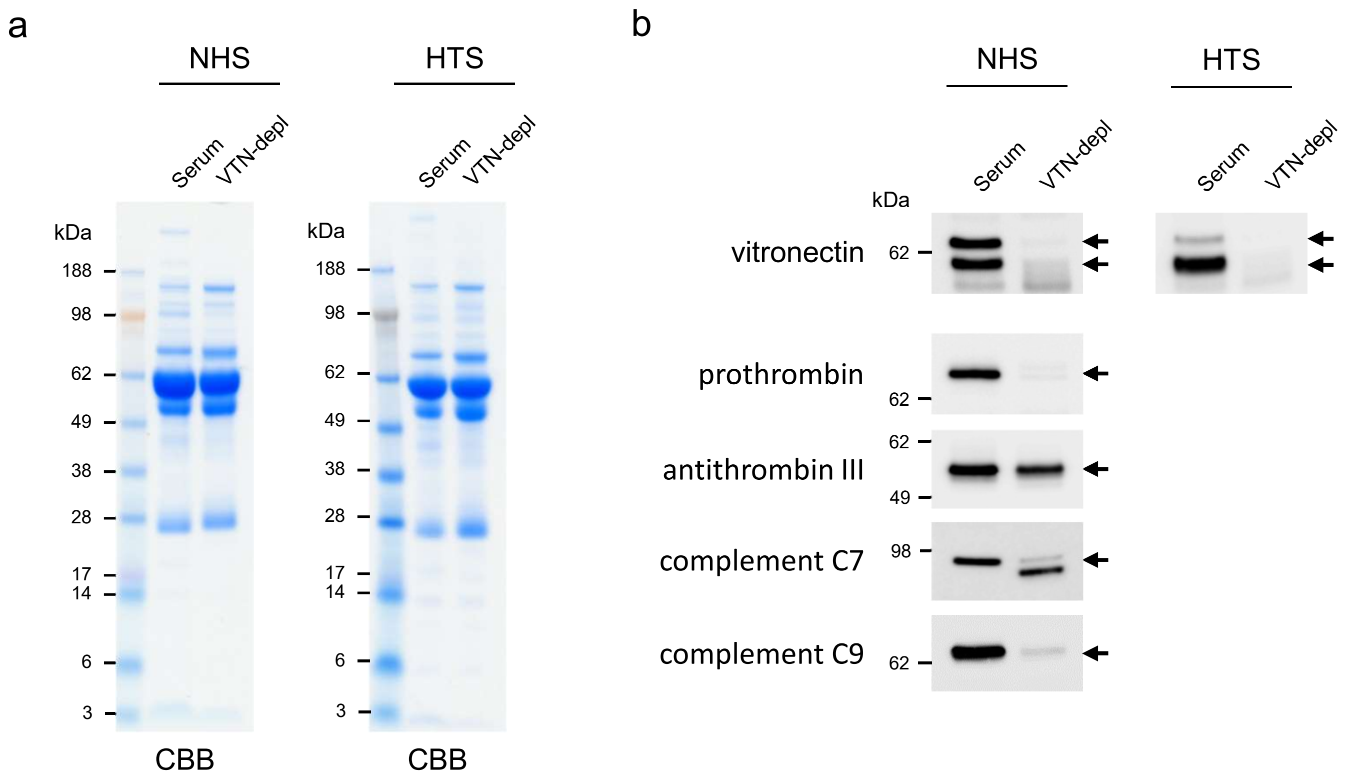

Supplementary Figure 10. Confirmation of binding of SE36 and VTN to the latex beads, Related to Figure 5

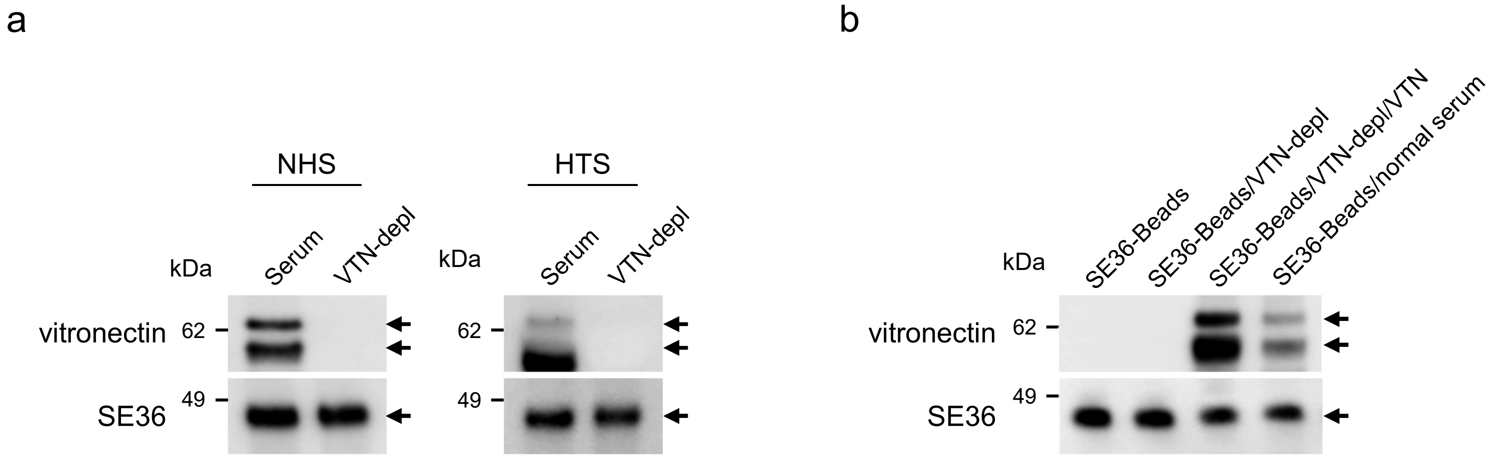

Supplementary Figure 11. Confirmation of presence of VTN in both of NHS and HTS, Related to Figure 5

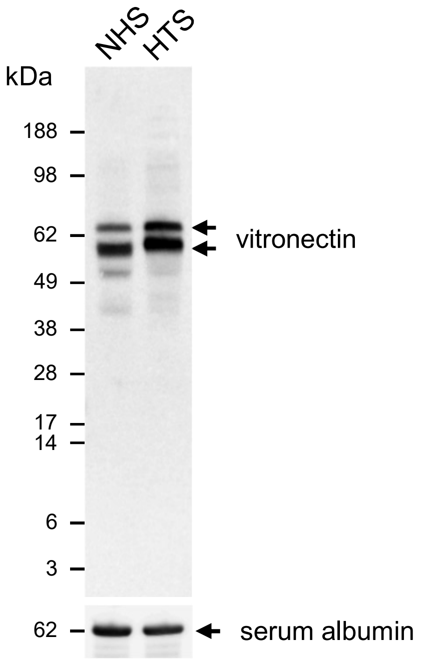

Supplementary Figure 12. Full Blots to Figure 3c

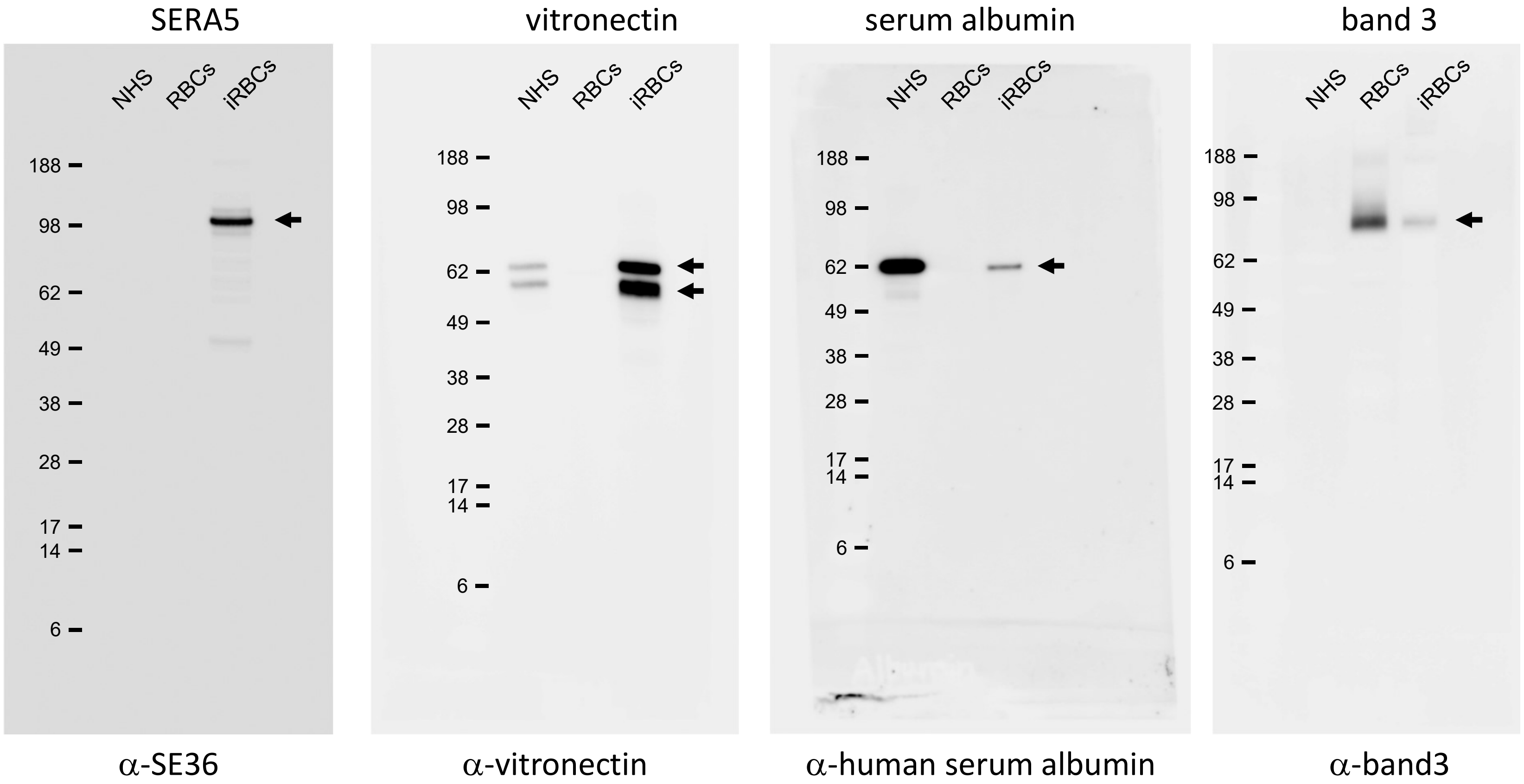

Supplementary Figure 13. Full Blots to Figure 5a(i)

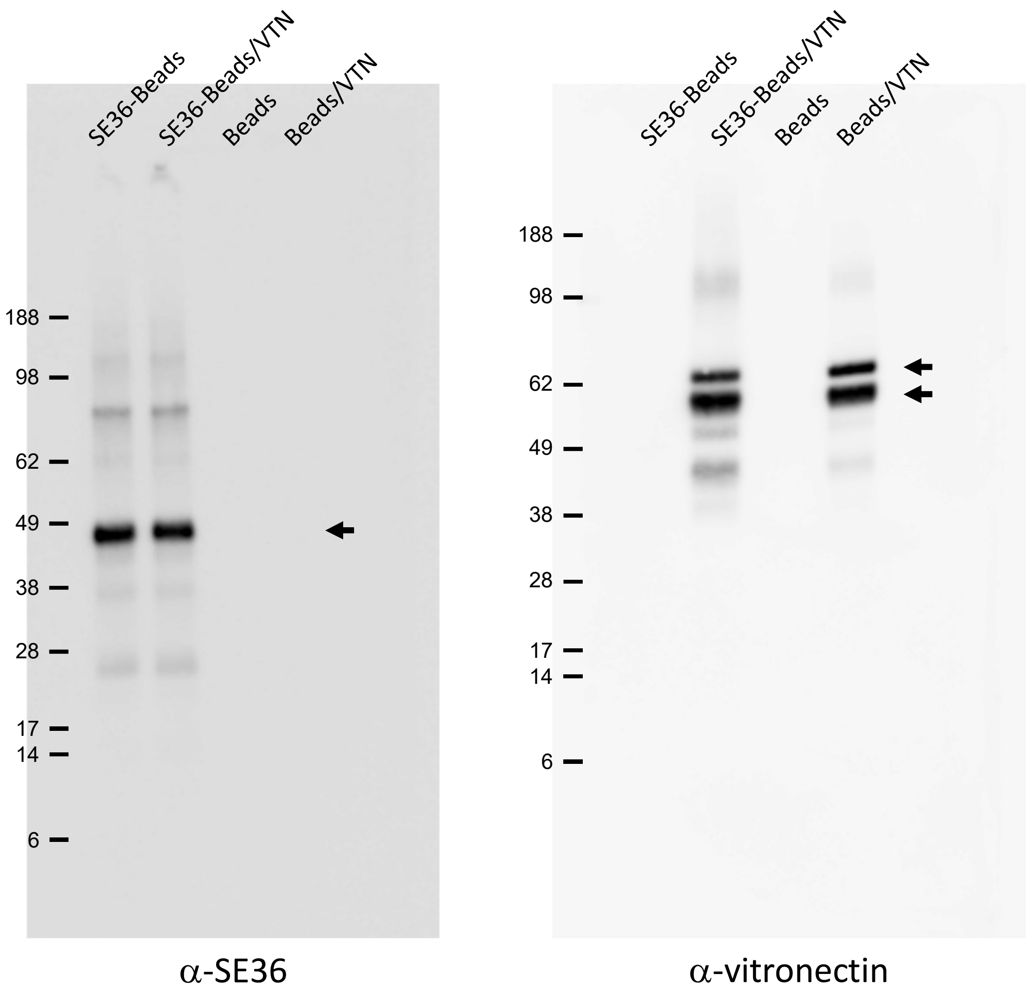

Supplementary Figure 14. Full Blots to Supplementary Figure 5a

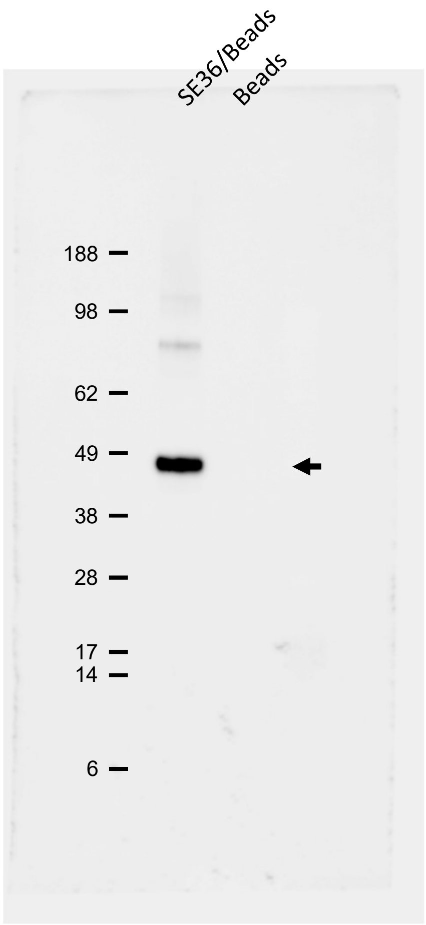

α-SE36

Supplementary Figure 15. Full Blots to Supplementary Figure 6a

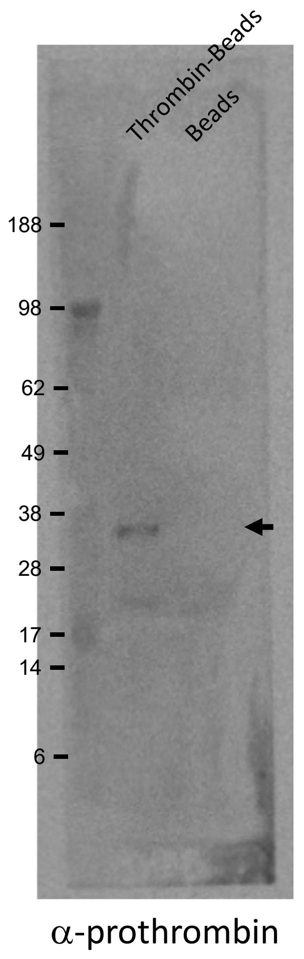

Supplementary Figure 16. Full Blots to Supplementary Figure 8a

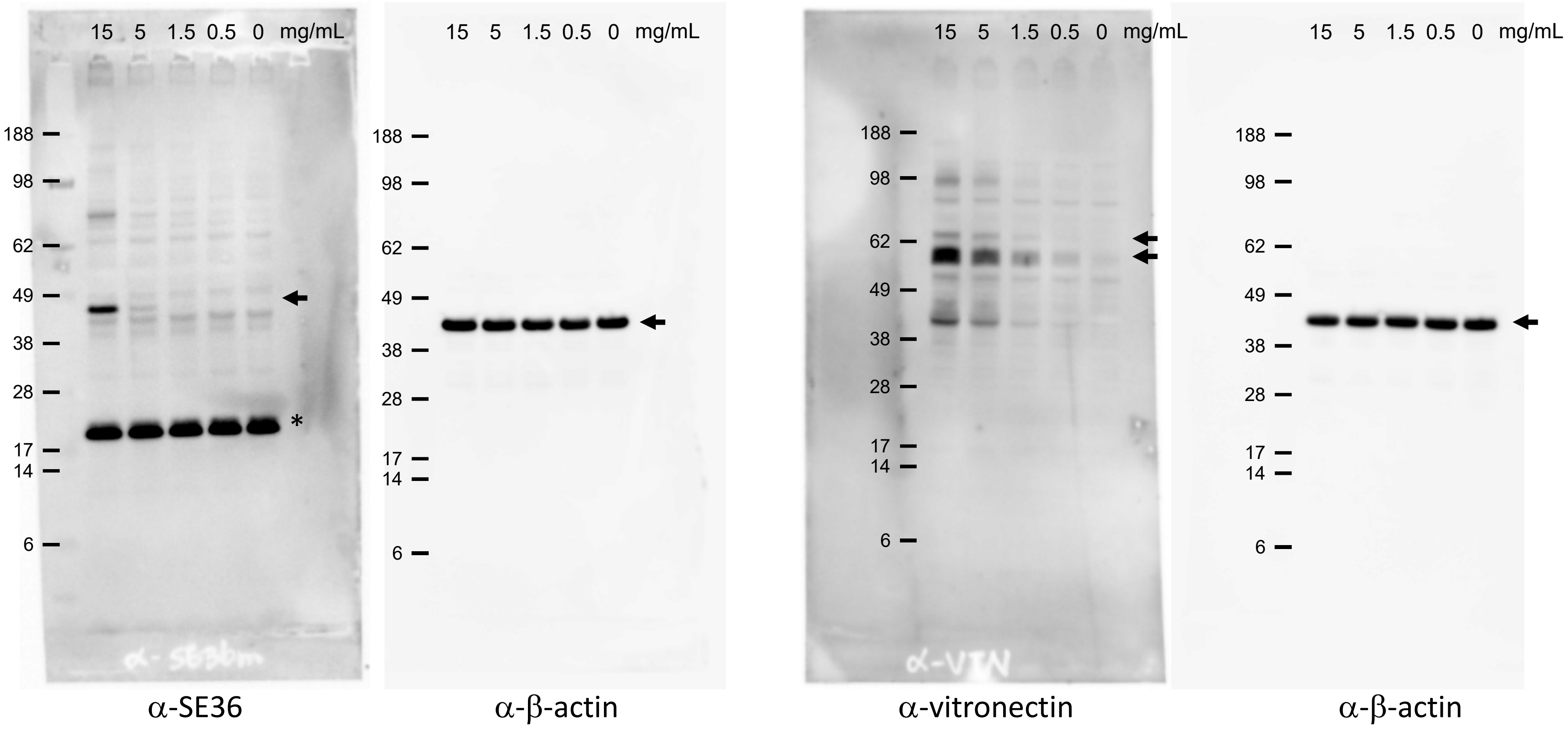

* non-specific band

Supplementary Figure 17. Full Blots to Supplementary Figure 8b

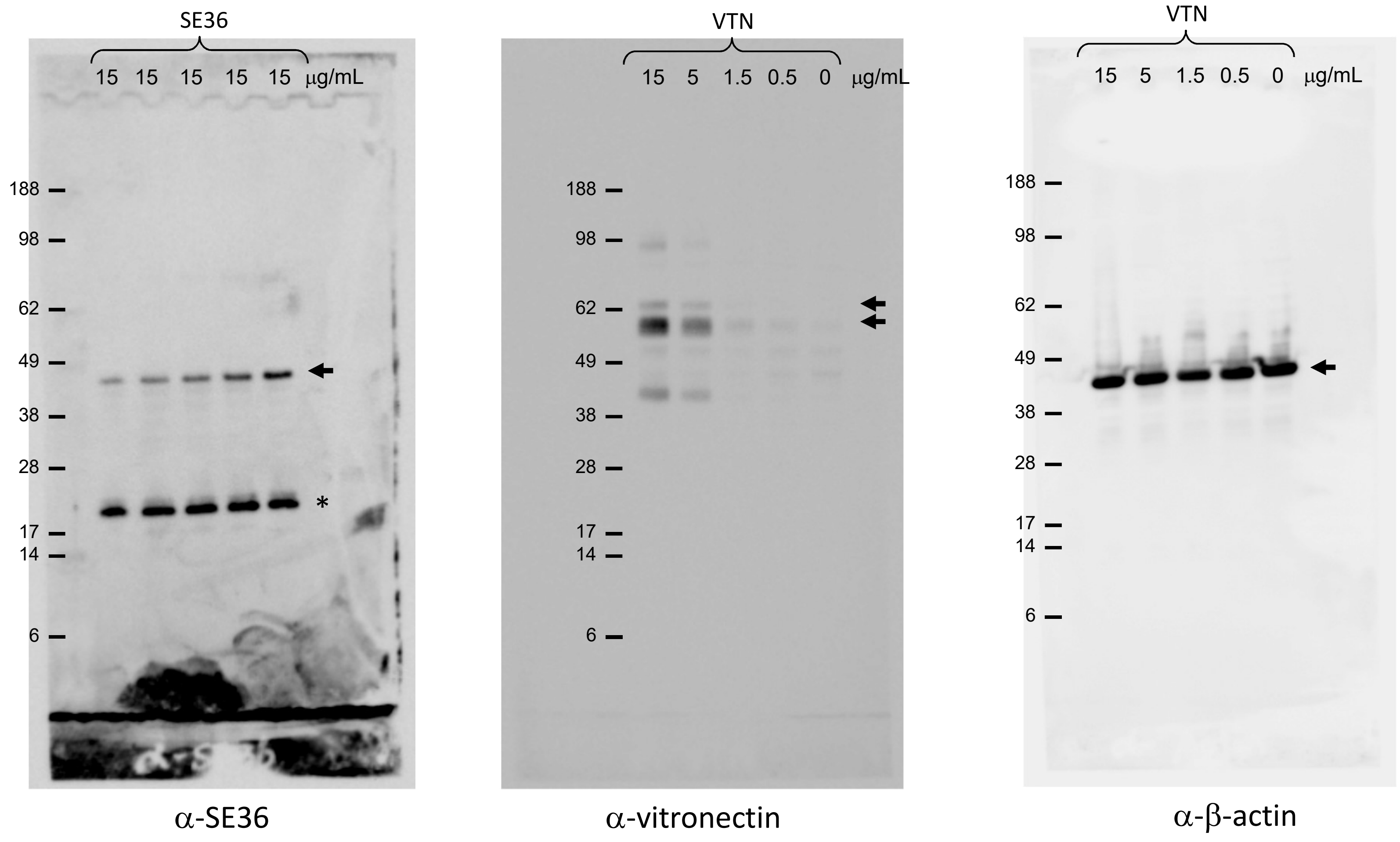

* non-specific band

Supplementary Figure 18. Full Blots to Supplementary Figure 9b

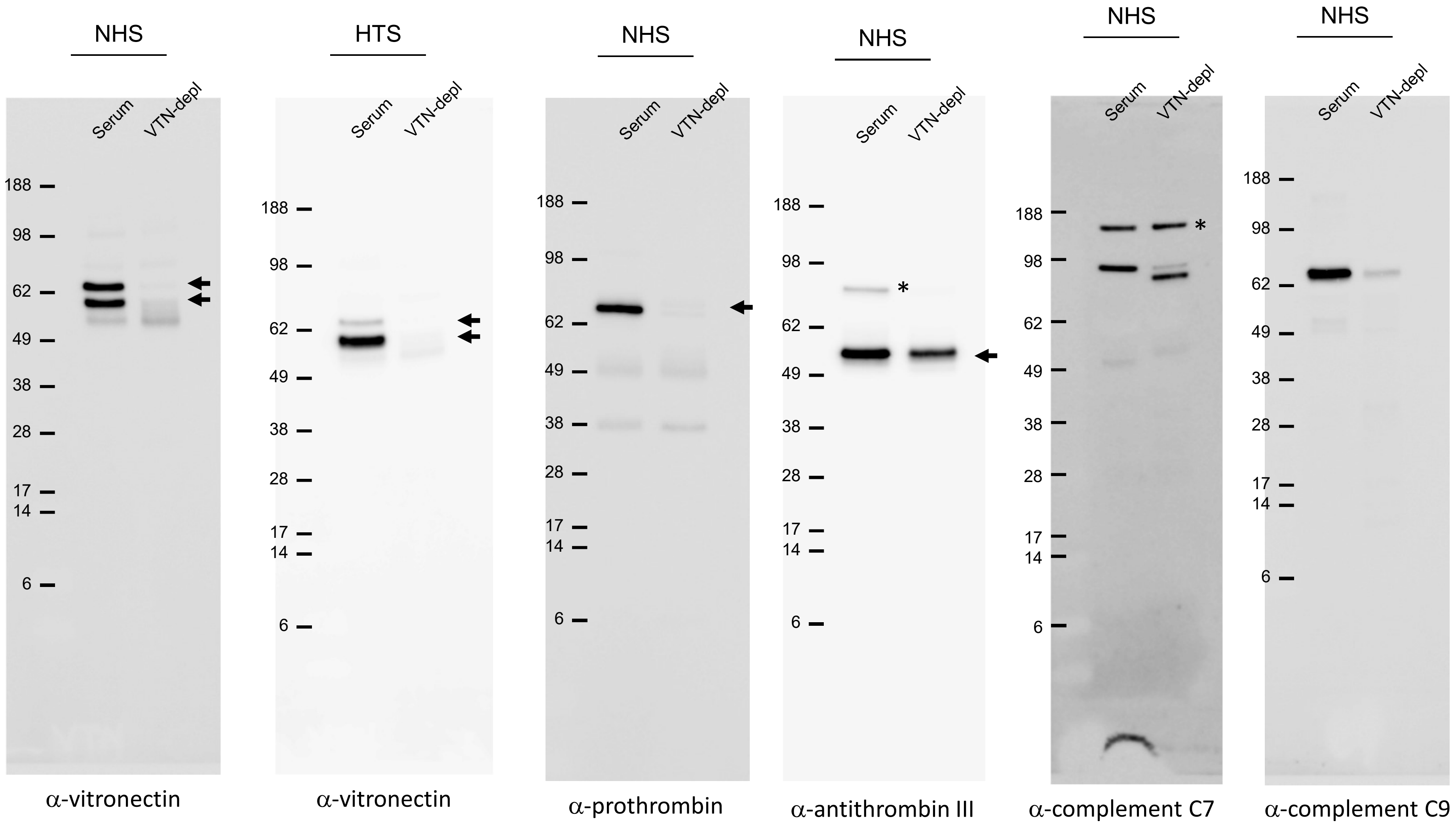

* non-specific band

Supplementary Figure 19. Full Blots to Supplementary Figure 10a

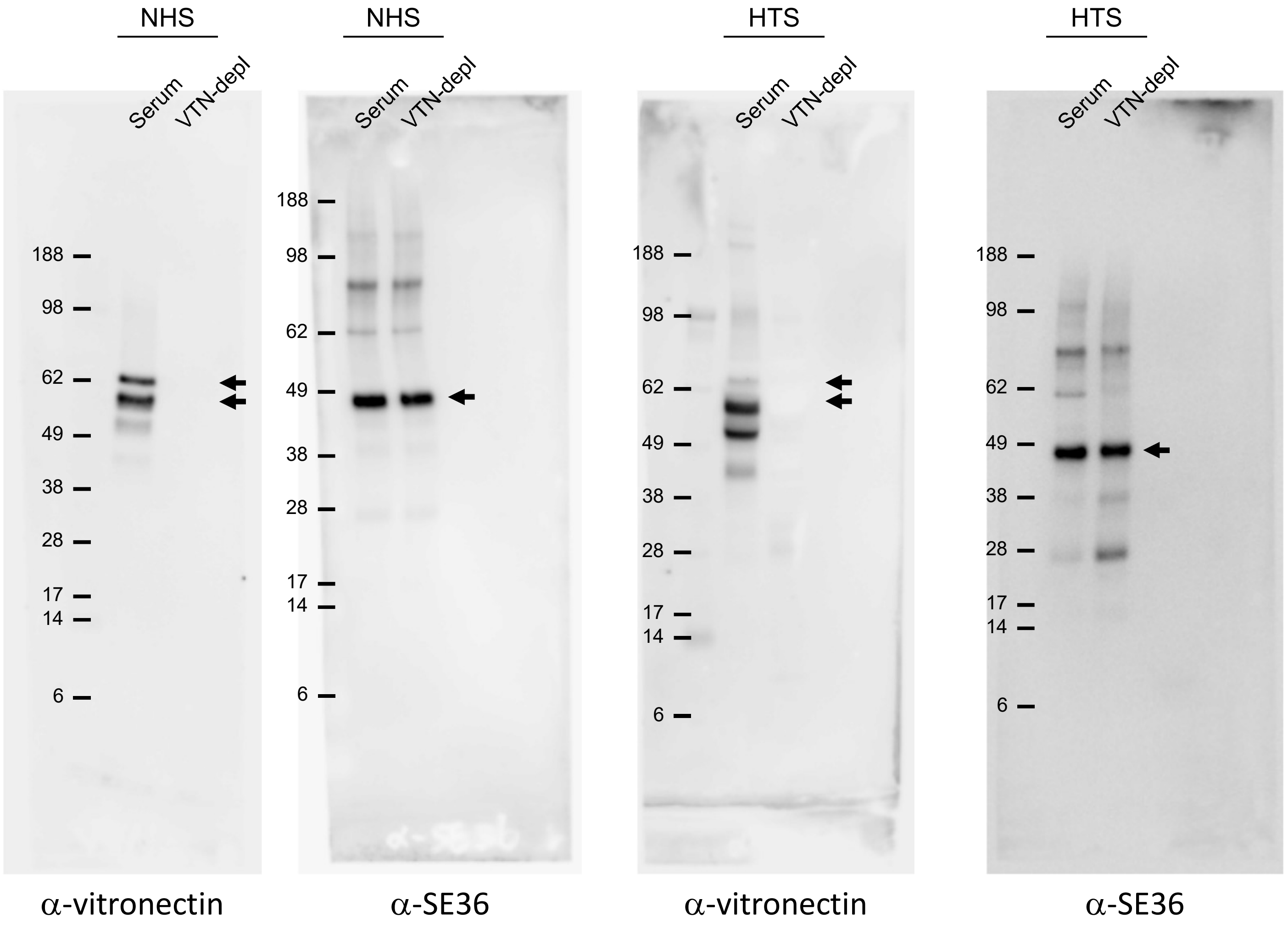

Supplementary Figure 20. Full Blots to Supplementary Figure 10b

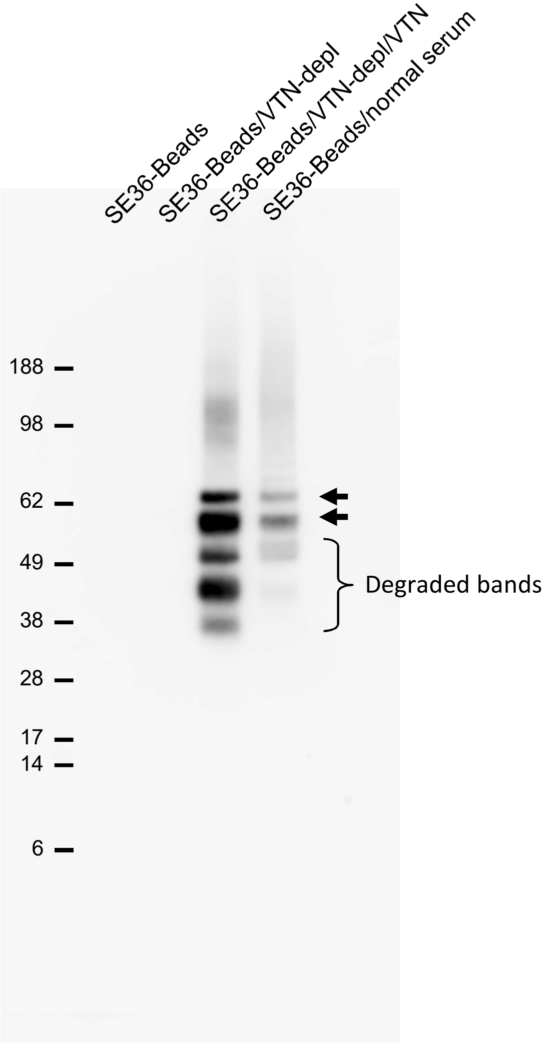

α-vitronectin

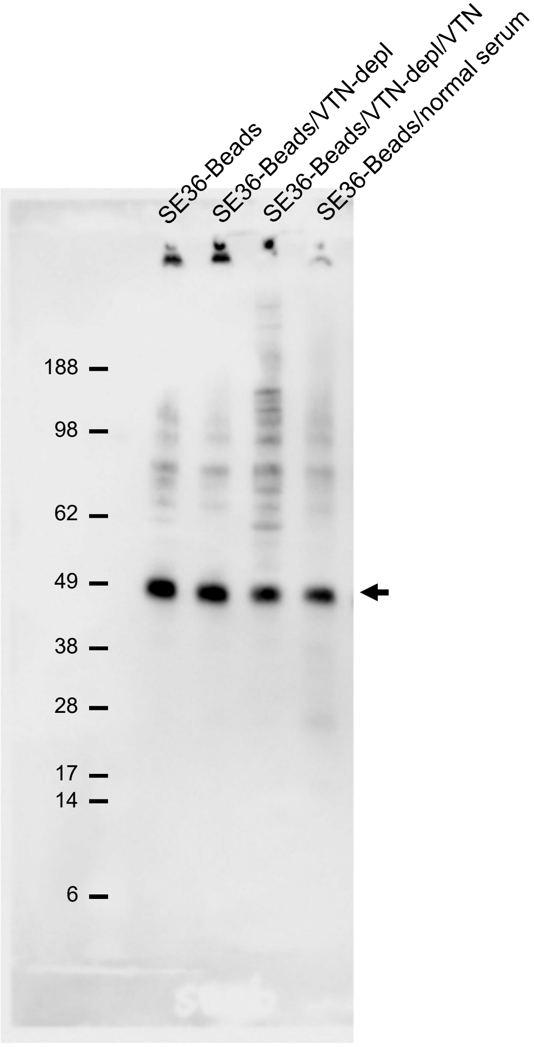

α-SE36

Supplementary Figure 21. Full Blots to Supplementary Figure 11

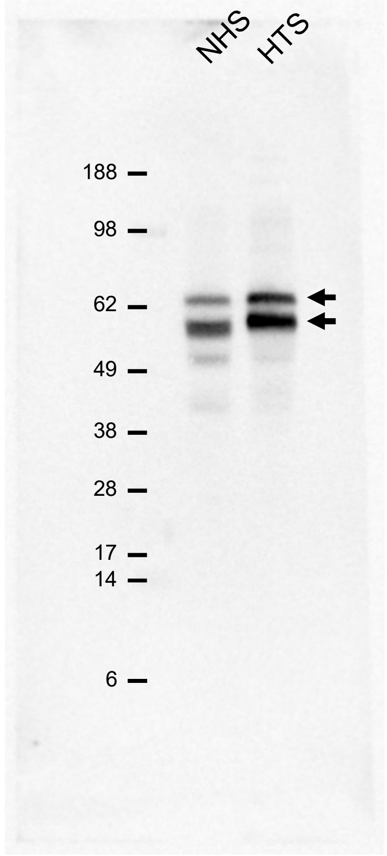

α-vitronectin

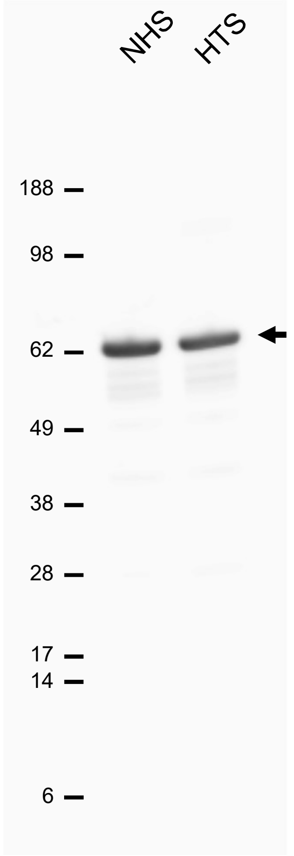

α-human serum albumin

Supplementary Table 1. Identification of SE36 binding proteins by shotgun LC-MS/MS analysis, Related to Figure 1

#	Protein name	SE36*	Control*	SE36/Control
1	Complement C5	12	0	–
2	Pregnancy-specific beta-1-glycoprotein 1	7	0	–
3	Filamin-A	6	0	–
4	Pregnancy-specific beta-1-glycoprotein 4	5	0	–
5	Cofilin-1	4	0	–
6	Actin, cytoplasmic 1	3	0	–
7	Extracellular matrix protein 1	3	0	–
8	Biotinidase	2	0	–
9	Coagulation factor XIII B chain	2	0	–
10	Sulfhydryl oxidase 1	2	0	–
11	CD5 antigen-like	26	1	26.00
12	Monocyte differentiation antigen CD14	11	1	11.00
13	Peroxiredoxin-2	8	1	8.00
14	Phosphatidylinositol-glycan-specific phospholipase D	15	2	7.50
15	Kallistatin	25	4	6.25
16	Carboxypeptidase B2	11	2	5.50
17	Complement component C8 alpha chain	5	1	5.00
18	Apolipoprotein B-100	724	157	4.61
19	Plasma kallikrein	18	4	4.50
20	Complement component C8 beta chain	25	6	4.17
21	Complement component C8 gamma chain	12	3	4.00
22	Phospholipid transfer protein	15	4	3.75
23	Proteoglycan 4	7	2	3.50
24	Apolipoprotein C-IV	6	2	3.00
25	Cathelicidin antimicrobial peptide	3	1	3.00
26	Fibronectin	443	149	2.97
27	Plasminogen	32	12	2.67
28	Apolipoprotein C-II	13	5	2.60
29	Lipopolysaccharide-binding protein	26	11	2.36
30	Fetuin-B	9	4	2.25
31	Histidine-rich glycoprotein	42	19	2.21
32	Apolipoprotein A-I	403	187	2.16
33	Prothrombin	158	76	2.08
34	Lumican	32	16	2.00
35	Complement component C7	20	10	2.00
36	Fibrinogen alpha chain	2	1	2.00
37	Thrombospondin-1	15	8	1.88
38	Heparin cofactor 2	49	27	1.81
39	Apolipoprotein C-I	7	4	1.75
40	Apolipoprotein L1	36	21	1.71
41	Pregnancy zone protein	56	33	1.70
42	Glutathione peroxidase 3	8	5	1.60
43	Complement component C9	44	29	1.52
44	Apolipoprotein A-IV	27	18	1.50
45	Mannan-binding lectin serine protease 2	6	4	1.50
46	Vitronectin	142	96	1.48
47	Antithrombin-III	130	90	1.44
48	Complement factor H	13	9	1.44
49	Inter-alpha-trypsin inhibitor heavy chain H4	56	40	1.40
50	Platelet factor 4	14	10	1.40
51	Protein Z-dependent protease inhibitor	7	5	1.40
52	Alpha-2-macroglobulin	223	168	1.33
53	von Willebrand factor	21	16	1.31
54	Complement C1r subcomponent	72	56	1.29
55	Ceruloplasmin	163	130	1.25
56	Complement C1s subcomponent	33	27	1.22
57	Alpha-1-antitrypsin	167	138	1.21
58	Haptoglobin-related protein	73	61	1.2
59	Hemopexin	38	32	1.19
60	Hemoglobin subunit beta	58	49	1.18
61	Gelsolin	91	77	1.18
62	Apolipoprotein E	54	46	1.17
63	Serum albumin	417	364	1.15
64	Serum paraoxonase/arylesterase 1	114	101	1.13
65	Complement C4-B	84	77	1.09
66	Sex hormone-binding globulin	16	15	1.07

* indicates total spectrum count (TSC) of proteins from each column.
Binding ability of serum proteins that bind to SE36 was calculated following the formula:
SE36/Control ratio = TSC of protein from SE36 column/TSC of protein from Control column

Supplementary Table 2. Molecules and antibodies used for ELISA-based binding assay, Related to Supplementary Figure 3

	Symbol	Product name	Catalog #	Manufacturer	Dilution
Molecule	VTN	Vitronectin, Human	G5381	Promega	2 μg/mL
	C5	Complement C5 from human serum	C3160	Sigma-Aldrich	2 μg/mL
	C7	Complement C7 from human serum	C2787	Sigma-Aldrich	2 μg/mL
	C8	Complement C8 from human serum	C3535	Sigma-Aldrich	2 μg/mL
	C9	Complement C9 from human serum	C3660	Sigma-Aldrich	2 μg/mL
	fH	Complement factor H from human plasma	C5813	Sigma-Aldrich	2 μg/mL
	ApoAI	Apolipoprotein A-I, Human, recombinant	019-20731	Wako	2 μg/mL
	HDL	High Density Lipoprotein, human	LP3	Merck	2 μg/mL
	LDL	LDL, human	LP2	Merck	2 μg/mL
	THRB	Recombinant Human Coagulation Factor II/Thrombin Protein, CF	1473-SE-010	R&D systems	2 μg/mL
	CLU	Recombinant Human Clusterin Protein, CF	2937-HS-050	R&D systems	2 μg/mL
	FINC	Human Fibronectin Protein, CF	1918-FN	R&D systems	2 μg/mL
Primary antibody	anti-VTN	Vitronectin clone 8E6(LJ8)	MAB88917	Millipore (mAb/mouse)*	1:2000
	anti-C5	C5/C5b	ab46153	Abcam (pAb/rabbit)	1:2000
	anti-C7	C7	ab126786	Abcam (mAb/rabbit)	1:2000
	anti-C8	C8	ab59140	Abcam (mAb/mouse)	1:2000
	anti-C9	C9	ab168345	Abcam (mAb/rabbit)	1:2000
	anti-fH	Factor H	ab133536	Abcam (mAb/rabbit)	1:2000
	anti-ApoAI	Apolipoprotein A1	ab7613	Abcam (pAb/goat)	1:2000
	anti-HDL	HDL	ab157595	Abcam (pAb/chicken)	1:2000
	anti-LDL	LDL	ab157795	Abcam (pAb/chicken)	1:2000
	anti-THRB	F2 Polyclonal (Thrombin)	24295-1-AP	Proteintech (pAb/rabbit)	1:2000
	anti-CLU	Human Clusterin Antibody	MAB2937	R&D systems (mAb/mouse)	1:2000
	anti-FINC	Fibronectin	ab32419	Abcam (mAb/rabbit)	1:2000
	anti-CD5L	CD5L (CD5 antigen-like)	ab45408	Abcam (pAb/rabbit)	1:2000
	anti-CD14	CD14 (Monocyte differentiation antigen CD14)	ab133335	Abcam (mAb/rabbit)	1:2000
Secondary antibody	anti-mouse	Peroxidase AffiniPure Goat Anti-Mouse IgG (H+L)	115-035-166	Jackson ImmunoResearch Laboratories	1:10000
	anti-rabbit	Peroxidase AffiniPure Donkey Anti-Rabbit IgG (H+L)	711-035-152	Jackson ImmunoResearch Laboratories	1:10000
	anti-Goat	Peroxidase AffiniPure Bovine Anti-Goat IgG (H+L)	805-035-180	Jackson ImmunoResearch Laboratories	1:10000
	anti-chicken	Peroxidase AffiniPure $F(ab')_2$ Fragment Donkey Anti-Chicken IgY (IgG) (H+L)	703-036-155	Jackson ImmunoResearch Laboratories	1:10000

* (Clonality/Host species)
